# Integrated analysis of the relationship between metabolic pathways and immune infiltration in rheumatoid arthritis

**DOI:** 10.3389/fimmu.2025.1679356

**Published:** 2025-12-12

**Authors:** Chaofeng Zhang, Zhongyi You, Zhiqun Pan, Yuming Huang, Zhiming Zhang, Qi Lin

**Affiliations:** 1 Department of Rheumatology and Immunology, the Affiliated Hospital of Putian University, Putian, Fujian, China; 2 School of Basic Medical Science, Putian University, Putian, Fujian, China; 3 Department of Rheumatology and Immunology, the Second Affiliated Hospital of Fujian Traditional Chinese Medical University, Fuzhou, Fujian, China; 4 Department of Pharmacy, the Affiliated Hospital of Putian University, Putian, Fujian, China; 5 Key Laboratory of Pharmaceutical Analysis and Laboratory Medicine, Putian University, Putian, Fujian, China

**Keywords:** COX7C, immune infiltration, machine learning, metabolic pathways, rheumatoid arthritis, TCA cycle

## Abstract

**Background:**

Rheumatoid arthritis (RA) is a chronic autoimmune disease characterized by persistent synovitis and systemic inflammation. Growing evidence highlights the critical role of metabolic dysregulation in RA pathogenesis, particularly through its impact on immune infiltration. However, the relationship between metabolic pathways and immune infiltration in RA remains unclear.

**Methods:**

This study integrated RNA-Seq data and clinical information from the GEO database to investigate the activity of seven major metabolic pathways and immune cell infiltration in RA patients. A co-expression network associated with RA was constructed using WGCNA, and key hub genes were identified using the LASSO logistic regression. A LASSO-based diagnostic model was then developed and validated across multiple independent datasets. To investigate the subcellular localization of the hub genes, scRNA-seq analysis was performed using publicly available datasets. Further correlation analyses were conducted to evaluate their involvement in immune infiltration and related metabolic processes. Additionally, the role of a key risk gene, COX7C, in RA was investigated through *in vivo* experiments.

**Results:**

Three dysregulated metabolic pathways, including amino acid, energy, and TCA cycle, were found to distinguish RA from healthy controls. A co-expression network strongly correlated with the TCA cycle was extracted. Then, eleven key hub genes were identified and appeared to bridge metabolic reprogramming and immune dysregulation. The LASSO model incorporating these genes showed robust diagnostic performance and correlated with RA disease severity. The scRNA-seq analysis revealed dysregulation of amino acid, lipid, carbohydrate, nucleotide, and TCA cycle metabolism in PBMCs from RA patients. COX7C was positively associated with amino acid and TCA cycle pathways in both PBMCs and fibroblasts, and was significantly upregulated in RA synovial tissue *in vivo*. Moreover, several drug compounds were identified as potentially effective by targeting COX7C.

**Conclusion:**

Metabolic pathways, particularly the TCA cycle, play a critical role in the pathogenesis of RA. Eleven key hub genes were identified as being involved in metabolic reprogramming associated with immune infiltration, and the LASSO model incorporating these genes demonstrated strong diagnostic potential for RA. Notably, COX7C might contribute to RA progression by dysregulating cellular metabolic processes.

## 1 Introduction

Rheumatoid arthritis (RA) is a chronic autoimmune disorder characterized by progressive and erosive joint inflammation (1, 2). With a global prevalence of 0.5%–1%, RA leads to functional disability in approximately 20% of patients within 1–5 years of disease onset, severely impairing quality of life and imposing a significant socioeconomic burden (2). Current diagnostic strategies primarily depend on serological biomarkers, including rheumatoid factor (RF) and antibody against the cyclic citrullinated protein (ACPA). However, the suboptimal sensitivity and specificity of these markers often result in delayed diagnosis and treatment, underscoring the need for more reliable biomarkers. Emerging evidence highlights systemic metabolic dysregulation in RA, particularly involving glucose, lipid, and amino acid metabolism (3, 4). Elucidating the mechanisms underlying metabolic dysregulation in RA may pave the way for novel diagnostic and therapeutic strategies targeting metabolic pathways.

Notably, emerging evidence has revealed that significant correlations between disease activity and altered levels of metabolites, such as 3-hydroxybutyrate, lactate, and urea, in both the blood and synovial fluid of RA patients (5). These metabolic alterations are associated with disease severity, inflammatory markers, and immune cell profiles, suggesting that dysregulated metabolic pathways, including glycolysis, tricarboxylic acid (TCA) cycle, amino acid metabolism, and fatty acid oxidation (FAO), collectively contribute to RA pathogenesis (4, 6, 7). The synovial microenvironment in RA exhibits metabolic aberrations reminiscent of tumor tissues, characterized by immune infiltration and heightened energy demands required to sustain cellular activation (2). Consequently, fibroblast-like synoviocytes (FLS) and immune cells undergo a metabolic shift from mitochondrial oxidative phosphorylation (OXPHOS) to adaptive glycolysis, accompanied by increased choline uptake and disrupted amino acid and lipid metabolism. This metabolic reprogramming leads to the pathological accumulation of TCA cycle intermediates, which act as inflammatory signaling molecules that amplify pro-inflammatory pathways, ultimately promoting sustained cartilage and bone destruction (2, 8). Despite some progress, the current understanding of RA heterogeneity from a metabolic perspective remains unclear.

Indeed, the inflammatory process in RA extends beyond the synovial tissue, exerting systemic effects on the entire organism (9). Current studies suggest that shared metabolic pathways, such as glycolysis and TCA cycle, contribute not only to local synovial damage but also to the systemic complications frequently observed in RA (10, 11). However, previous studies have mostly focused on individual metabolic enzymes or relying on bulk tissue analyses without considering shared metabolic pathways (5, 12). A comprehensive assessment of metabolic pathway alterations and their association with immune infiltration across the entire body remains lacking. This study aimed to characterize metabolic reprogramming in RA by systematically analyzing both blood and synovial tissue using integrative bioinformatics approaches. Key genes involved in metabolic dysregulation were identified, and their biological functions were explored. Moreover, this study highlights the diagnostic significance of altered metabolic pathways in RA and proposes critical metabolic pathway related genes as potential diagnostic biomarkers and novel therapeutic targets for disease intervention.

## 2 Materials and methods

### 2.1 Data preprocessing

Standardized RNA-Seq data and corresponding clinical information for RA samples from the GSE89408 (13), GSE93272 (14), and GSE93776 (14) datasets were obtained from the Gene Expression Omnibus (GEO, https://www.ncbi.nlm.nih.gov/geo/; **Supplementary Table S1**). Single-cell RNA sequencing (scRNA-Seq) data used in this study were retrieved from the dataset published by Binvignat et al. (15), available through the CellxGene Discover platform (https://cellxgene.cziscience.com/collections/e1a9ca56-f2ee-435d-980a-4f49ab7a952b). GSE109449 dataset (16) included gene expression profile of synovial fibroblasts from 2 RA patients. As previous studies (17, 18), a total of 1,916 metabolism-related gene sets including seven major metabolic pathways (amino acid, carbohydrate, energy, lipid, nucleotide, TCA cycle, and vitamin cofactor metabolism) which annotated based on the REACTOME database (https://reactome.org; **Supplementary Table S2**) were collected.

### 2.2 Gene set variation analysis

To evaluate the activity of the major metabolism-related gene sets in the GSE89408 (13) and GSE93272 (14) datasets, gene set variation analysis (GSVA) was performed for individual gene sets across all samples using GSVA package (19). Differential gene set scores between RA samples and healthy controls (HCs) were compared using the limma package (20). The discriminatory performance of each gene set was further assessed using receiver operating characteristic (ROC) curve analyses.

### 2.3 Immune infiltration analysis

To investigate the potential role of immune infiltration in the pathogenesis of RA, the proportions of 22 immune cell types in the GSE89408 (13) and GSE93272 (14) datasets were estimated using the CIBERSORT algorithm (21). This analyses, which infers immune cell fractions from bulk RNA transcriptomic data, was performed on the CIBERSORTx website using the LM22 gene signature matrix and in strict adherence to the recommended guidelines. Correlation analyses were then performed between the metabolic enrichment scores and immune cell infiltration levels.

### 2.4 Weighted gene co-expression network analysis

Given that metabolic dysregulation is a key driver of RA (6), the weighted gene co-expression network analysis (WGCNA) (22) was applied to investigate gene modules significantly associated with metabolism-related gene sets. Co-expression networks were constructed to reveal modules with the strongest correlations to the target metabolic pathways.

### 2.5 Identification of key hub genes

Differentially expressed genes (DEGs) were identified using the limma package (20) and intersected with genes from the relevant modules to pinpoint potential hub genes. These candidate hub genes were subsequently analyzed using least absolute shrinkage and selection operator (LASSO) logistic regression with the glmnet package (23) to identify the key hub genes, and the performance was assessed via nested cross-validation.

### 2.6 Functional enrichment analysis

To investigate the biological functions of the key hub genes, Gene Ontology (GO) enrichment analyses and Kyoto Encyclopedia of Genes and Genomes (KEGG) and REACTOME pathway analyses were performed using the Database for Annotation, Visualization and Integrated Discovery (DAVID; https://davidbioinformatics.nih.gov) (24). To further evaluate the involvement of these genes in specific signaling pathways and biological processes, Gene Set Enrichment analyses (GSEA) was conducted via the WEB-based Gene SeT analyses Toolkit (WebGestalt, http://www.webgestalt.org/#) (25). Additionally, to explore potential interactions among the proteins encoded by the hub genes, a protein–protein interaction (PPI) network was constructed using GeneMANIA (https://genemania.org) (26).

### 2.7 Prediction of potential therapeutic drug

Potential therapeutic drugs targeting the key hub genes were predicted using the Drug Signatures Database (DSigDB) via Enrichr (https://maayanlab.cloud/Enrichr), with an adjusted p-value < 0.05 set as the significance threshold. The structures of the candidate drugs were obtained from the simplified molecular input line entry system (SMILES), and the PDB identifier for the key gene was retrieved from the UniProt database (https://www.uniprot.org). Subsequently, molecular docking analyses was performed using PyMOL (Version 3.1.3, Schrödinger).

### 2.8 Construction and validation of diagnostic model

Subsequently, key hub genes were evaluated using ten machine learning models, including Boosted Trees, C5.0 rules, Decision Tree, LASSO Logistic Regression, Logistic Regression, Multilayer Perceptron, Naive Bayes, Nearest Neighbor, Random Forest, and Support Vector Machine-Recursive Feature Elimination (SVM-RFE). The optimal diagnostic model was selected based on the highest area under the ROC curve (AUC) and was additionally assessed by calibration curves and decision curve analysis. The diagnostic score was calculated using the following formula: Diagnostic model value = Σ (ki × Expi), where ki represents the coefficient of gene i and Expi denotes its expression level. The diagnostic model values were compared between RA samples and HCs across multiple datasets, and ROC curves were generated to assess diagnostic performance. Additionally, correlations between the diagnostic model scores, key hub gene expression, and immune cell infiltration were analyzed. A nomogram based on the optimal diagnostic model was constructed and evaluated using the rms package.

### 2.9 ScRNA-seq analysis

The scRNA-seq data were processed and analyzed following the standard workflow of the Seurat package (version 4.4.0) (27). Quality control filtering for the scRNA-seq data included retaining genes detected in at least 5 cells and cells expressing a minimum of 100 genes. Cells with high mitochondrial content (>25%) were excluded from further analyses. Potential doublets were identified and removed using the Scrublet package. Normalization was performed followed by Seurat integration to correct for batch effects across samples, and clustered for cell annotation. The scRNA-seq dataset from the publication by Binvignat et al. (15), which includes peripheral blood mononuclear cell (PBMC) samples from 18 RA patients and 18 healthy controls, enrolled 21,946 genes and 108,609 cells. Meanwhile, 191 fibroblasts obtained from GSE109449 dataset (16). Next, the metabolism related gene sets enrichment scores were performed using GSVA package (19).

### 2.10 Collagen-induced arthritis rat model and histopathological analysis

As previous studies (28, 29), Wistar rats (6–8 weeks old, weighing 180 ± 20 g) were purchased from Shanghai SLAC Laboratory Animal Co., China (Batch: SCXK (Hu) 2017–0005). On day 0, each rat received a subcutaneous injection of 200 μL of an emulsion composed of immunization-grade bovine type II collagen (CII, Chondrex, USA) mixed with incomplete Freund’s adjuvant (IFA, Chondrex, USA) at the base of the tail. A booster injection of the same emulsion (200 μL) was administered in the same manner on day 7. Arthritis was allowed to develop for 28 days. On the other hand, normal saline (NS; Sichuan Kelun, China) was injected as the control group (CON). Following successful induction of the CIA model, synovial joint tissues were harvested and subjected to histopathological evaluation. Hematoxylin and eosin (H&E) staining (Beyotime, China) and safranin O staining (Yuanye, China) were performed according to the manufacturers’ protocols and observed under a microscope (Eclipse C1, Nikon, Japan). Immunohistochemistry (IHC) staining was performed as previously described (28). A rabbit polyclonal antibody against COX7C (Cat. 11411-2-AP, Proteintech, China) was used to stain synovial tissue sections. This study was approved by the Ethics Committee of the Affiliated Hospital of Putian University (Approval ID: 202130).

### 2.11 Statistical analysis

All data analyses were performed using R programming (version 4.4.1). Differences between two groups were assessed using either Student’s t-test or Wilcoxon rank-sum test, depending on data distribution. For comparisons involving more than two groups, one-way ANOVA or the Kruskal–Wallis test was applied. Correlations between variables were evaluated using Spearman’s correlation analyses. A p-value < 0.05 was considered statistically significant.

## 3 Results

### 3.1 Identification of key metabolic pathways in RA

**Figure 1:**
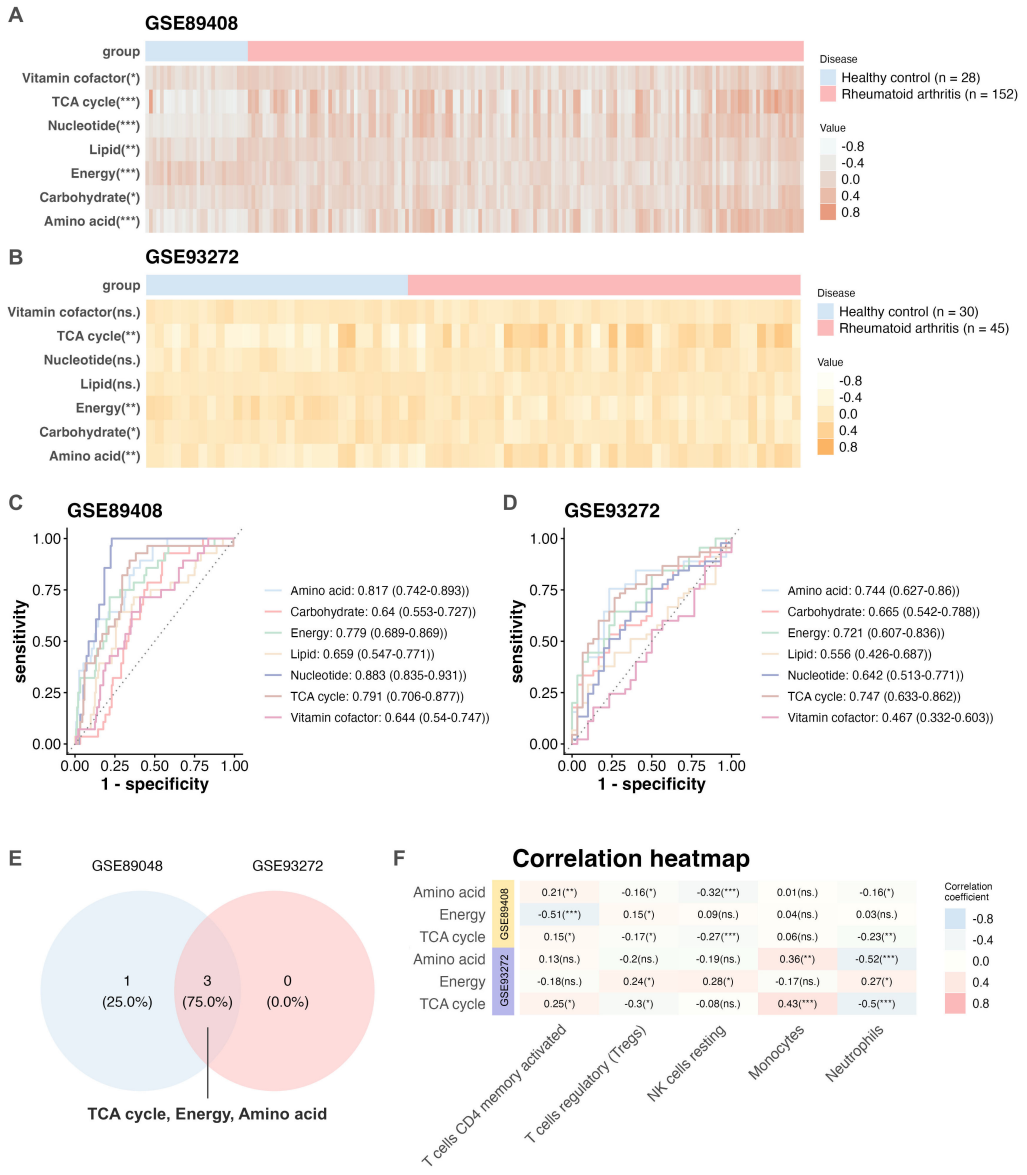
Identification of key metabolic pathways in ra. Heatmaps showing differences between RA samples and HCs across seven metabolic pathways using the GSE89408 **(A)** and GSE93272 **(B)** datasets. ROC curves and corresponding AUC values evaluating the predictive accuracy of the seven metabolic pathways in the GSE89408 **(C)** and GSE93272 **(D)** datasets. **(E)** Based on AUC values > 0.7 in both datasets, three metabolic pathways were identified and selected for further analysis. **(F)** Heatmaps illustrating the correlation between the selected three metabolic pathways and immune cell infiltration using the GSE89408 and GSE93272 datasets. AUC, area under the ROC curve; GSE, Gene Expression Omnibus Series; HC, healthy control; RA, rheumatoid arthritis; ROC, receiver operating characteristic. ***p < 0.001; **p < 0.01; *p < 0.05; ns, not significant.

The GSE89408 (13) and GSE93272 (14) datasets were included in this study. The GSE89408 (13) was normalized using the edgeR package (30), whereas GSE93272 was analyzed using the original expression matrix obtained from the GEO database. Data quality and normalization were assessed using boxplots (**Supplementary Figures S1A, B**), and PCA plots further confirmed the successful mitigation of batch effects (**Supplementary Figures S1C, D**). RA progression is characterized by significant dysregulation of multiple metabolic pathways (3, 6). Using the GSVA method (19), we quantified the activity of seven major metabolic pathways in arthritis patients (n = 190) compared to HCs (n = 28) within the GSE89408 dataset (13). Compared to HCs, arthritis patients exhibited upregulation of lipid, nucleotide, TCA cycle, and amino acid metabolism, alongside downregulation of energy integration pathways (**Supplementary Figure S2A**). Subsequently, RA samples compared to HCs across both the GSE89408 (13) and GSE93272 (14) datasets. As shown in **Figure 1A**, analyses of the GSE89408 dataset (13) revealed that RA synovial tissue (n = 152) exhibited significantly elevated activity in amino acid, nucleotide, and TCA cycle metabolism (p < 0.001), along with downregulation of energy metabolism, compared to normal tissue (n = 28). Similar trends were observed in whole blood transcriptomes from the GSE93272 dataset (HCs: n = 30; RA: n = 45) (14), with amino acid, energy, and TCA cycle showing relative dysregulation in RA (**Figure 1B**, **Supplementary Table S3**). ROC curve analysis (**Figures 1C, D**, **Supplementary Table S4**) across both the GSE89408 (13) and GSE93272 (14) datasets revealed that dysregulated metabolic pathways—including amino acid, energy, and TCA cycle metabolism—displayed strong diagnostic potential (AUC > 0.7; **Figure 1E**). Complementary immune cell infiltration analyses via CIBERSORT (21) filtering out immune cell subsets deemed absent (CIBERSORT p-value ≥ 0.05) in over 80% of the samples across both cohorts. the results identified significant upregulation of CD4+ memory T cells activated, T cells regulatory (Tregs), NK cells resting, monocytes, and neutrophils in both datasets (**Supplementary Figures S2B–D**). Notably, we found a potential positive correlation between TCA cycle metabolism and the immune infiltration of CD4+ memory T cells (**Figure 1F**), suggesting a mechanistic link between metabolic pathways and immune infiltration in RA.

### 3.2 Identification of co-expression module in RA

**Figure 2:**
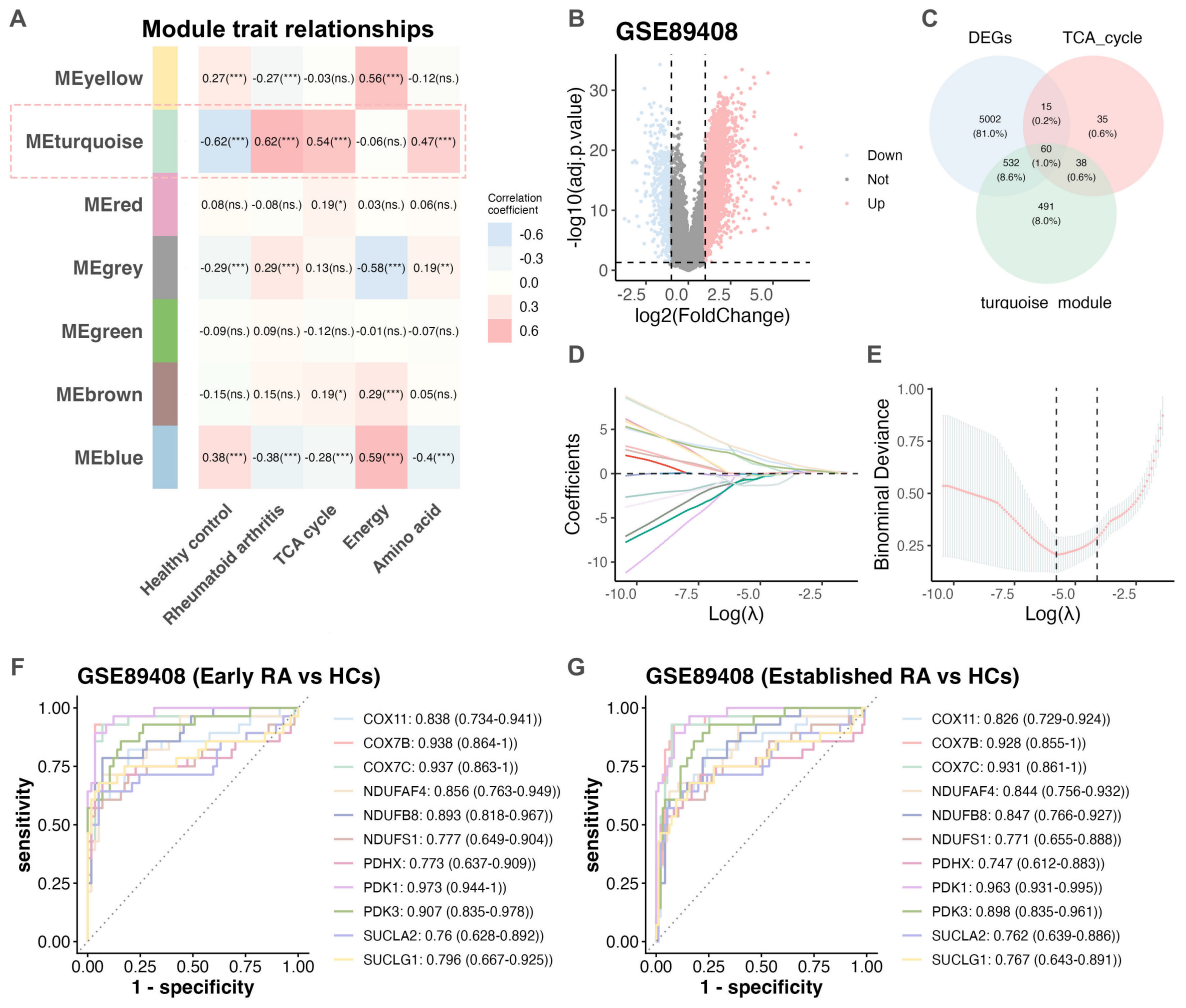
Identification of co-expression module and key hub genes in TCA cycle metabolism. **(A)** Heatmaps showing the correlation between differential modules identified by WGCNA and the three selected metabolic pathways. **(B)** Volcano plot of DEGs from the GSE89408 dataset (adjusted *p* < 0.05, |log_2_fold change (log_2_FC)| > 1). **(C)** Venn diagram showing the intersection of 60 genes among DEGs, TCA cycle–related genes, and genes within the turquoise module. **(D)** LASSO logistic regression used to select key genes associated with RA. **(E)** Eleven hub genes were selected by LASSO analysis, and the two dotted vertical lines indicate the optimal values based on the lambda.min and lambda.1se criteria. ROC curves and corresponding AUC values were used to assess the predictive performance of these genes in distinguishing early RA **(F)** and established RA **(G)** from HCs. AUC, area under the ROC curve; DEG, Differentially expressed gene; GSE, Gene Expression Omnibus Series; HC, healthy control; LASSO, least absolute shrinkage and selection operator; RA, rheumatoid arthritis; ROC, receiver operating characteristic; TCA, tricarboxylic acid; WGCNA, weighted gene co-expression network analysis. ***p < 0.001; **p < 0.01; *p < 0.05; ns, not significant.

To identify gene modules associated with RA, WGCNA (22) was performed using the GSE89408 dataset (13). A total of 2,176 genes related to metabolic pathways were retrieved from the REACTOME database (**Supplementary Table S5**). WGCNA (22) was conducted with a soft-thresholding power of β = 5 (**Supplementary Figures 3A, B**). The clustering dendrogram of metabolism-related genes is presented in **Supplementary Figure S3C**. Based on weighted correlations, hierarchical clustering identified seven distinct co-expression modules, each represented by a different color (**Figure 2A**). Subsequently, the turquoise module exhibited the strongest correlation with RA (r = 0.62, p < 0.001). Further analyses revealed that this module (n = 1,121; **Supplementary Table S6**) was significantly associated with key metabolic pathways, including amino acid (r = 0.47, p < 0.001), and TCA cycle metabolism (r = 0.54, p < 0.001; **Figure 2A**). These findings suggest that the turquoise module represents a major RA-related co-expression network enriched in metabolic processes.

### 3.3 Identification of key hub genes in the TCA cycle metabolism

Differential expression analyses of the GSE89408 dataset (13) using the limma package (20) (with adjusted p < 0.05, |Log2 fold change (log2FC)| > 1) identified 5,609 DEGs between RA samples and HCs (**Figure 2B**, **Supplementary Table S7**). Notably, previous studies have reported significant alterations in TCA cycle metabolites within both immune cells and synovial tissue of RA patients (11, 31). WGCNA revealed that the turquoise module showed a strong positive (r > 0.5) correlation with TCA cycle metabolism (r = 0.54, p < 0.001; **Figure 2A**), containing 148 genes identified as TCA cycle–related (**Supplementary Table S2**). Through a three-way intersection of these TCA cycle–related genes, the DEGs, and the turquoise module, 60 overlapping candidate genes were identified (**Figure 2C**, **Supplementary Table S8**), and PPI network was presented in **Supplementary Figure S4**. Subsequently, LASSO logistic regression analyses (**Figures 2D, E**) refined this gene list to eleven hub genes: COX7C, COX7B, PDK1, PDK3, NDUFB8, COX11, NDUFAF4, SUCLG1, NDUFS1, PDHX, and SUCLA2. To obtain more realistic performance estimates and mitigate overfitting, we performed a nested cross-validation (10-fold outer and 5-fold inner loops) using the nestedcv package (32). The results showed that the nested cross-validation model mirrored the performance of our LASSO model, validating the robustness of our original approach (**Supplementary Figure S5A**). The expression levels of the eleven hub genes were significantly elevated in RA samples compared to HCs (p < 0.001; **Supplementary Figure S5B**), and exhibited strong diagnostic potential in distinguishing RA from HCs (AUC > 0.7; **Supplementary Figure S5C**, **Supplementary Table S9**). Among the 57 early RA and 95 established RA samples in GSE89408 dataset (13), the expression of these genes in two types of RA samples were significantly upregulated (p < 0.001; **Supplementary Figure S5D**). Notably, these genes demonstrated high diagnostic value in identifying both early and established RA (**Figures 2F, G**, **Supplementary Table S9**) in GSE89408 dataset (13), with COX7B, COX7C, and PDK1 achieving AUC values greater than 0.9, indicating strong discriminatory power. Correlation analyses revealed that most of these genes were positively associated with the TCA cycle, except for PDK3, PDK1, and COX11, which showed weaker correlations (r < 0.5). Additionally, several hub genes were correlated with CD4+ T cells and Tregs (**Supplementary Figure S5E**). These findings demonstrated that most of these genes might be involved in the reprogramming of the TCA cycle in RA.

### 3.4 Functional enrichment and potential therapeutic drug analysis of key hub genes

**Figure 3:**
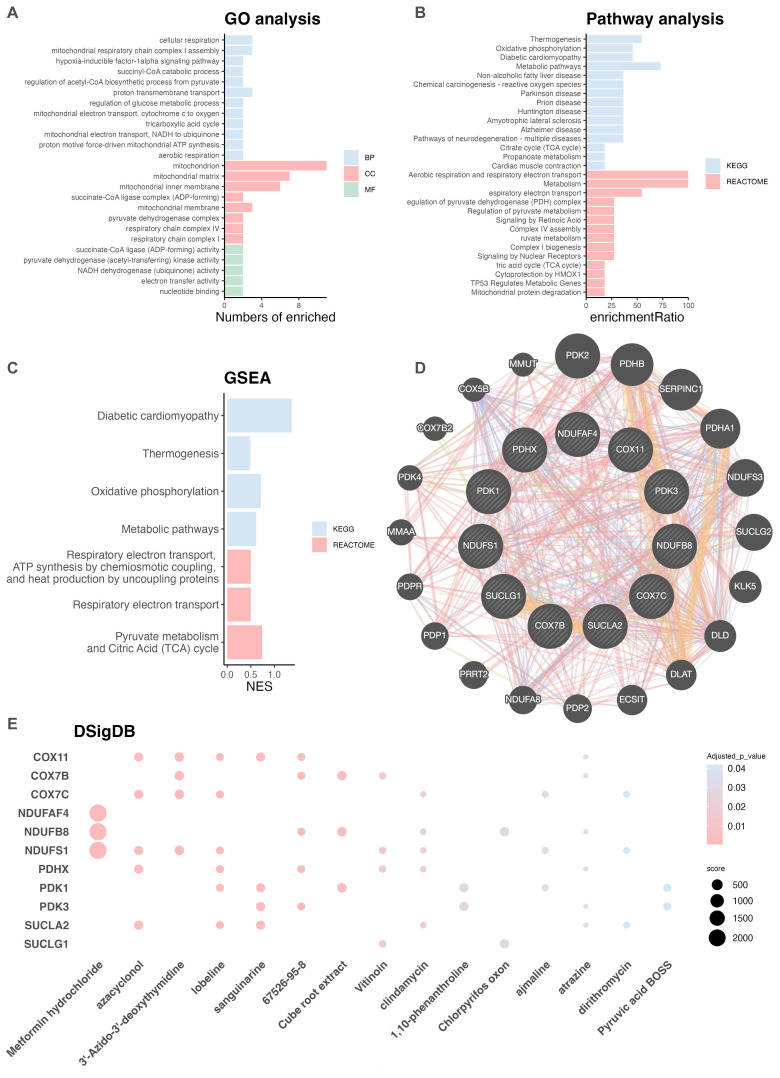
Functional enrichment analysis. **(A)** GO enrichment analysis of the eleven selected hub genes. **(B)** Pathway enrichment analysis of the eleven genes based on KEGG and Reactome databases. **(C)** GSEA of the eleven genes using the GSE89408 dataset. **(D)** PPI network constructed using GeneMANIA. **(E)** Drug sensitivity analysis identified fifteen candidate compounds targeting the hub genes, based on the DSigDB database via Enrichr. DSigDB, Drug Signatures Database; GO, Gene Ontology; GSE, Gene Expression Omnibus Series; GSEA, Gene set enrichment analysis; KEGG, Kyoto Encyclopedia of Genes and Genomes; PPI, protein–protein interaction.

To further elucidate the potential biological functions of the eleven key hub genes, GO and KEGG pathway enrichment analyses were performed using the DAVID database (24). As shown in **Figure 3A** (**Supplementary Table S10**), GO enrichment identified eleven significantly associated biological processes (BP), including cellular respiration, mitochondrial respiratory chain complex I assembly, and proton transmembrane transport. Eight cellular component (CC) terms revealed that these genes were predominantly localized to the mitochondrion, mitochondrial matrix, and mitochondrial inner membrane. Molecular function (MF) enrichment analyses identified five terms, notably including electron transfer activity, nucleotide binding and pyruvate dehydrogenase kinase activity. The KEGG (n = 15) and REACTOME (n = 14) pathway analyses (**Figure 3B**, **Supplementary Table S11**) demonstrated that the identified genes were primarily involved in metabolic pathways, aerobic respiration and respiratory electron transport. Specifically, a substantial proportion of the identified genes, including COX7C, COX7B, and NDUFS1, were involved in the dysregulation of mitochondrial oxidative phosphorylation. To assess the dyregulation of these pathways in RA, GSEA was conducted on the GSE89408 dataset (13) based on log2FC values. The results indicated that pyruvate metabolism and TCA cycle were significantly dysregulated in RA patients (**Figure 3C**, **Supplementary Table S12**). Furthermore, to explore the potential interactions among these genes, a PPI network was constructed using GeneMANIA (26), as shown in **Figure 3D**. Through integration of the DSigDB dataset using Enrichr, 15 drug compounds (adjusted p < 0.05; **Supplementary Table S13**), such as metformin hydrochloride, sanguinarine, azacyclonol, and 3’-Azido-3’-deoxythymidine were identified as significantly associated with the key hub genes with high combined scores, as shown in **Figure 3E**.

### 3.5 Construction and validation of a metabolism−related diagnostic model

**Figure 4:**
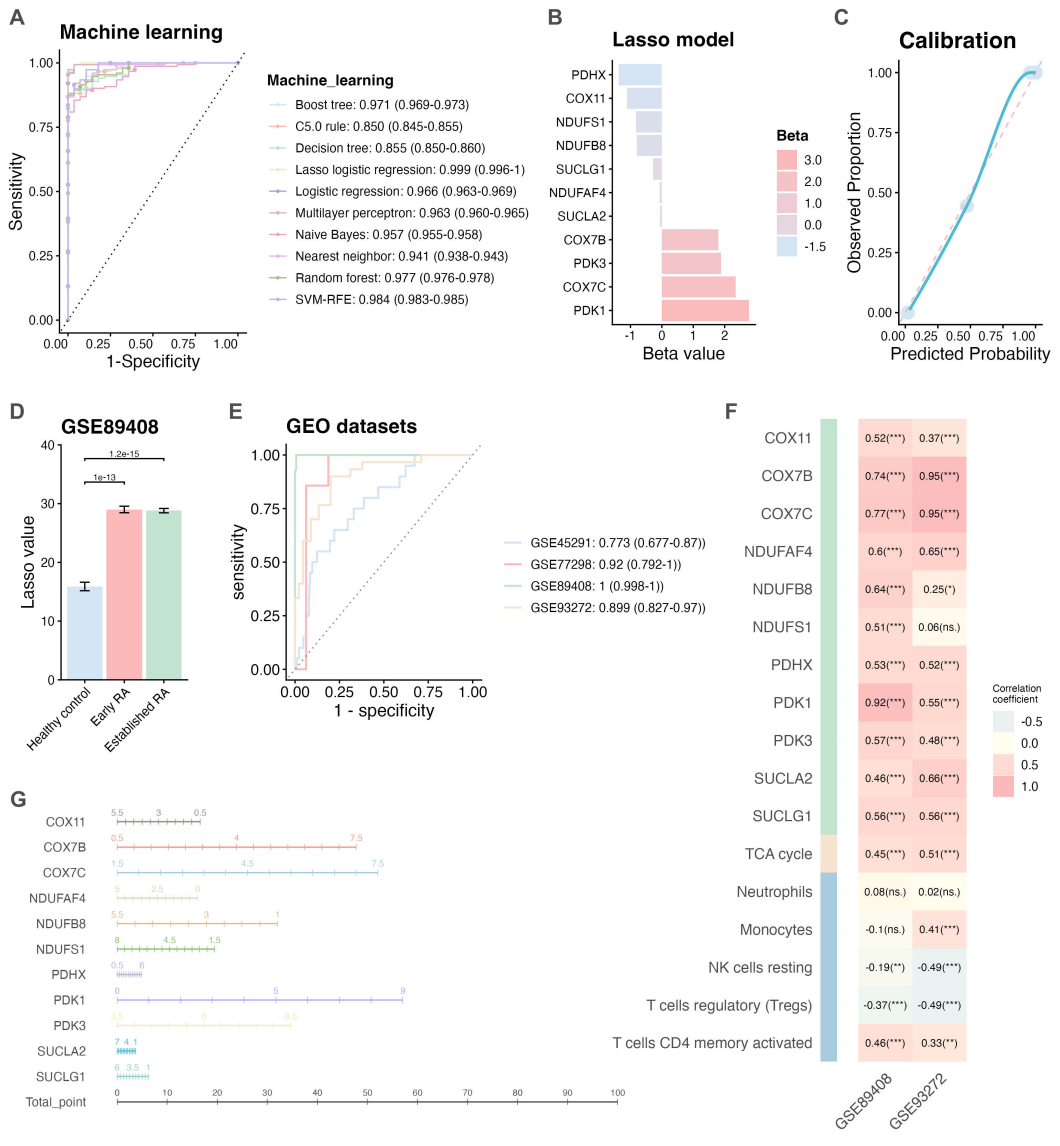
Construction and validation of a metabolism-related diagnostic model. **(A)** Ten machine learning algorithms, including Boosted Trees, Decision Tree, LASSO Logistic Regression, Logistic Regression, Multilayer Perceptron, Naive Bayes, Nearest Neighbor, Random Forest, C5.0 Rules, and Support Vector Machine-Recursive Feature Elimination, were applied. ROC curves display the AUC values for each model. **(B)** The LASSO model was identified as the optimal classifier, with beta coefficients of selected genes shown in the bar plot. **(C)** Calibration plot showed excellent agreement between predicted probabilities and observed outcomes. **(D)** Expression levels of the LASSO model score among early RA, established RA, and HCs in the GSE89408 dataset. **(E)** ROC curves and corresponding AUC values evaluating the predictive performance of the LASSO model in distinguishing RA from HCs across multiple datasets (GSE45291, GSE772098, GSE89408, and GSE93272). **(F)** Heatmaps showing the correlations between the LASSO model values, key hub genes, TCA cycle activity, and immune cell infiltration based on the GSE89408 and GSE93272 datasets. **(G)** Nomogram plot based on the GSE89408 dataset illustrating the predictive contribution of key hub genes. AUC, area under the ROC curve; GSE, Gene Expression Omnibus Series; HC, healthy control; LASSO, least absolute shrinkage and selection operator; RA, rheumatoid arthritis; ROC, receiver operating characteristic; TCA, tricarboxylic acid. ***p < 0.001; **p < 0.01; *p < 0.05; ns, not significant.

To further construct a metabolism-related diagnostic prediction tool, based on GSE89408 dataset (13), eleven key hub genes were enrolled into ten machine learning. The optimal diagnostic model was identified through comparing the auc value of ROC curve (**Supplementary Table S14**). as shown in **Figure 4A**, LASSO logistic regression was an optimal model (AUC = 0.999). As shown in **Figure 4B** (**Supplementary Table S15**), A Lasso model was: Lasso value = COX7C * 2.3558 + COX7B * 1.7983 + PDK1 * 2.7770 + PDK3 * 1.8816 + COX11 * -1.1158 + NDUFAF4 * -0.0719 + NDUFB8 * -0.7949 + NDUFS1 * -0.8306 + SUCLG1 * -0.2759 + PDHX * -1.3711 + SUCLA2 * -0.0634. Next, calibration plots demonstrated excellent agreement between predicted probabilities and observed outcomes (**Figure 4C**), with a Brier score of 0.0078 indicating good model calibration. We performed internal and external validation using the GSE89408 dataset (13), which yielded an external validation AUC of 0.991 (**Supplementary Figure S6A**), confirming the model’s generalizability. And decision curve analysis further confirms the clinical utility of our model across a range of threshold probabilities (**Supplementary Figure S6B**). The GSE89408 dataset (13), which includes synovial tissue samples from both early and established RA patients as well as HCs, showed significantly higher LASSO values in both early and established RA samples compared to HCs (p < 0.05; **Figure 4D**; **Supplementary Figure S6C**). Similarly, synovium from RA patients in the GSE77298 dataset (33) also exhibited significantly elevated LASSO values (p < 0.05; **Supplementary Figure S6D**). In whole blood samples, increased LASSO values were observed in RA patients in both the GSE93272 (14) (p < 0.05; **Supplementary Figure S6E**) and GSE45291 (34) (p < 0.05; **Supplementary Figure S6F**) datasets. ROC analyses indicated that all four GEO datasets yielded AUC values above 0.7 (**Figure 4E**, **Supplementary Table S16**), with the LASSO model demonstrating excellent diagnostic performance for both early and established RA (AUC > 0.99; **Supplementary Figure S6G**). As shown in **Figure 4F**, the expression of the key hub genes was strongly correlated with the LASSO model value in both the GSE89408 (13) and GSE93272 (14) datasets. Additionally, the LASSO model value was positively correlated with TCA cycle activity and CD4+ T cell memory activated. Finally, a nomogram model was constructed based on the expression levels of the key hub genes and the LASSO model value (**Figure 4G**). The model identified PDK1, COX7B, and COX7C as the primary contributors to the diagnostic potential in RA. Collectively, these results suggest that the diagnostic model based on the LASSO model value has robust predictive potential for identifying RA patients. Based on the GSE93272 dataset (14), the LASSO model values showed positive correlations with Matrix Metalloproteinase-3 (MMP3), Clinical Disease Activity Index (CDAI), Swollen Joint Count (SJC28), Disease Activity Visual Analog Scale (D.VAS), and Simplified Disease Activity Index (SDAI), suggesting that the model may reflect disease severity in RA (**Supplementary Figure S6H**).

### 3.6 Subcellular localization of key hub genes in RA and their association with metabolic dysregulation

**Figure 5:**
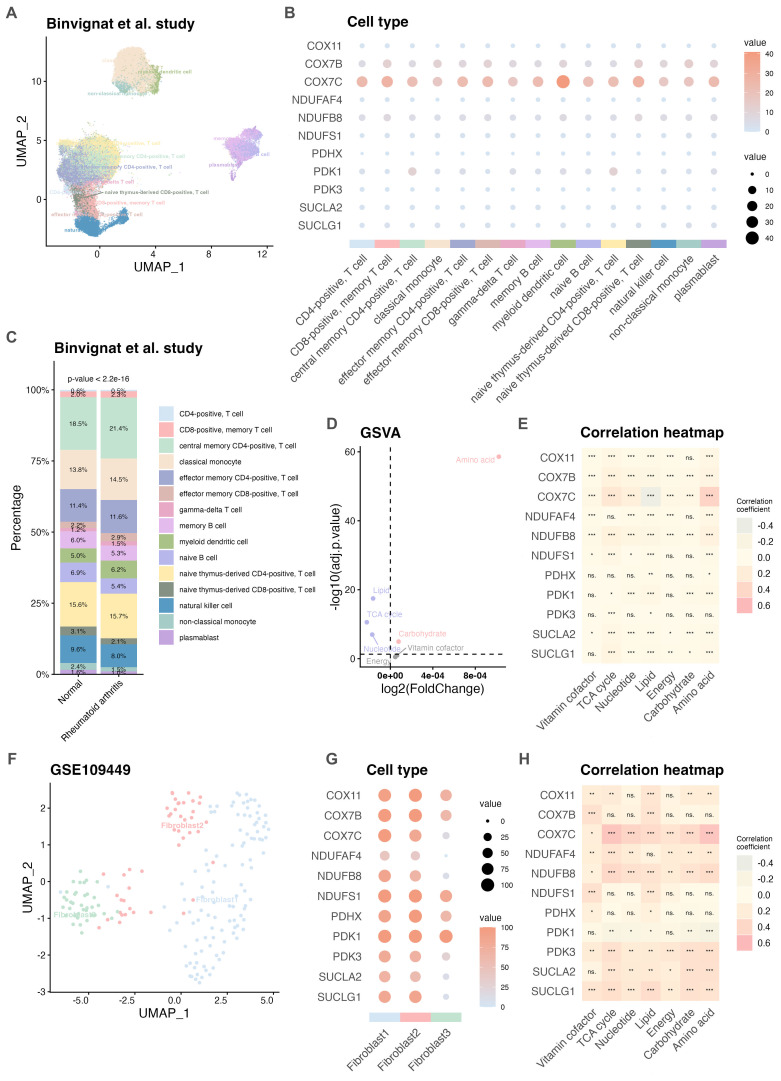
Subcellular localization of key hub genes in RA and their association with metabolic dysregulation. **(A)** UMAP plot showing the distribution of different cell types in the Binvignat et al. study. **(B)** Correlation between various cell types and key hub genes based on the Binvignat et al. study. **(C)** Comparison of cell type proportions between RA and HCs in PBMCs. **(D)** Differential analysis of seven metabolic pathways performed using the limma package based on the Binvignat et al. study. **(E)** Heatmap displaying the correlation between altered metabolic pathways and key hub genes based on the Binvignat et al. study. **(F)** UMAP plot showing distinct cell types in the GSE109449 dataset. **(G)** Correlation between different cell types and key hub genes in the GSE109449 dataset. **(H)** Heatmap showing the relationship between differential metabolic pathways and key hub genes based on GSE109449. GSE, Gene Expression Omnibus Series; HC, healthy control; PBMC, Peripheral blood mononuclear cell; RA, rheumatoid arthritis. ***p < 0.001; **p < 0.01; *p < 0.05; ns, not significant.

The identification of the subcellular localization of key hub genes provides insight into their potential molecular functions in RA. After quality control of the scRNA-seq data, approximately 108,609 high-quality cells from Binvignat et al. (15) were retained and categorized into fifteen distinct immune cell types (**Figure 5A**) as following the reference available at CellxGene (https://cellxgene.cziscience.com/), including classical monocyte, myeloid dendritic cell, central memory CD4+ T cell, CD8+ memory T cell, non-classical monocyte, natural killer (NK) cell, naive thymus-derived CD4+ T cell, effector memory CD4+ T cell, naive B cell, memory B cell, naive thymus-derived CD8+ T cell, gamma-delta T cell, plasmablast, effector memory CD8+ T cell, and CD4+ T cell. The expression patterns of the eleven key hub genes across these different cell types were analyzed (**Supplementary Figures S7**). As shown in **Figure 5B**, COX7C, COX7B, and PDK1 were broadly expressed across multiple cell types. Compared to normal PBMCs (n = 60,007), the proportion of various immune cell types showed significant differences in RA samples (n = 48,602; **Supplementary Figure S8A**). Particularly, the percentages of central memory CD4+ T cell were significantly higher in RA samples than in normal PBMCs (p < 0.05; **Figure 5C**), suggesting a potential role for these cell subsets in RA pathogenesis. The expression levels of key hub genes were also compared between RA and normal PBMCs (**Supplementary Figure S8B**) using the Wilcoxon rank-sum test. Furthermore, significant differences in cell cycle phase distribution were observed between RA samples and HCs (p < 0.05; **Supplementary Figure S8C**). And the involvement of the eleven key hub genes in the cell cycle was assessed. COX7B, COX7C, and NDUFB8 were predominantly enriched in the G1 phase, while PDK1 was more highly expressed in the G2/M and S phases (**Supplementary Figure S8D**). Subsequently, seven major metabolic pathways were evaluated in RA samples using the GSVA method. Except for energy and vitamin cofactor metabolism (p > 0.05), significant differences were observed in amino acid, lipid, carbohydrate, nucleotide, and TCA cycle metabolism between RA and normal PBMCs using limma package (20) (p < 0.05; **Figure 5D**, **Supplementary Figure S8E**). We found that the level of COX7C were positively correlated with amino acid and TCA cycle metabolism (p < 0.05; **Figure 5E**). Hyperplasia of synovial fibroblasts is one of the main contributors to synovial infiltration in RA (3). analyses of the GSE109449 dataset (16) identified 191 high-quality synovial fibroblasts from two RA patients, which clustered into three distinct modules (**Figure 5F**). Except for NDUFAF4, most key hub genes showed strong correlations with the three fibroblast modules (**Figure 5G**, **Supplementary Figure S8F**). Using the GSVA method, COX7C, NDUFB8, PDK3, and SUCLG1 were positively associated with metabolic dysregulation (**Figure 5H**). Collectively, these findings suggested that COX7C might serve as a key regulator of metabolic reprogramming in RA.

### 3.7 COX7C was associated with metabolic dysregulation in RA

**Figure 6:**
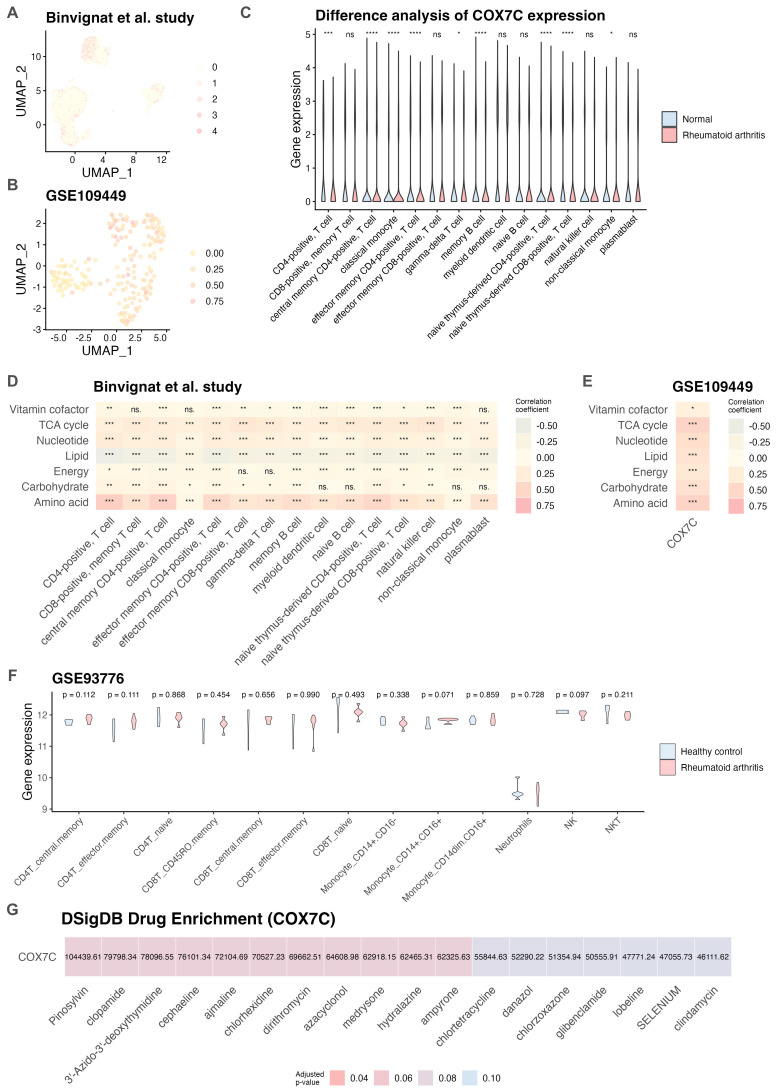
COX7C was associated with metabolic dysregulation in RA. **(A)** UMAP plot showing the distribution of COX7C in the Binvignat et al. study. **(B)** UMAP plot showing the distribution of COX7C in the GSE109449 dataset. **(C)** Differential expression analysis of COX7C across cell types in PBMCs based on the Binvignat et al. study. **(D)** Heatmap displaying the correlation between COX7C expression levels in different cell types and altered metabolic pathways based on the Binvignat et al. study. **(E)** Heatmap displaying the correlation between COX7C expression levels in fibroblast and altered metabolic pathways based on the GSE109449 dataset. **(F)** Differential expression analysis of COX7C across cell types based on the GSE93776 dataset. **(G)** Drug sensitivity analysis identified eighteen candidate compounds targeting COX7C, based on the DSigDB database via Enrichr. DSigDB, Drug Signatures Database; GSE, Gene Expression Omnibus Series; HC, healthy control; PBMC, Peripheral blood mononuclear cell; RA, rheumatoid arthritis. ***p < 0.001; **p < 0.01; *p < 0.05; ns, not significant.

**Figure 7:**
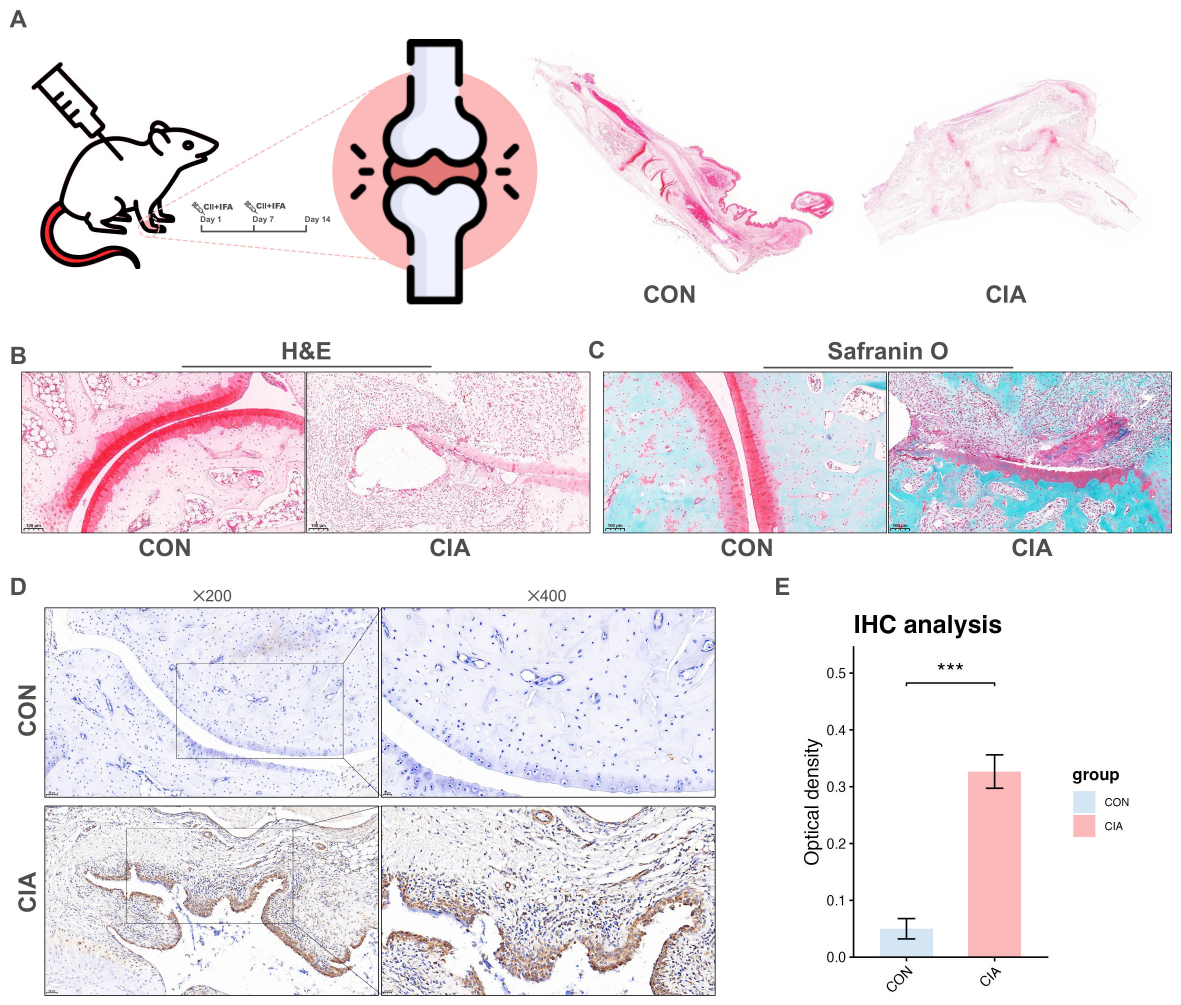
The upregulated COX7C expression in CIA rat model. **(A)** Study flowchart. **(B)** Representative images of joint and synovial tissue pathology shown through H&E staining; magnification × 100, scale bar: 100 μm; left, CON group; right, CIA group. **(C)** Representative images of joint and synovial tissue pathology shown through safranin O staining; magnification × 100, scale bar: 100 μm; left, CON group; right, CIA group. **(D)** Representative IHC images showing COX7C expression in joint and synovial tissues; Up: CON group, magnification × 200, scale bar: 50 μm, and modify, magnification × 400, scale bar: 20 μm. Bottom: CIA group, magnification × 200, scale bar: 50 μm, and modify, magnification × 400, scale bar: 20 μm. **(E)** Comparison of optical density of CIA and CON group. CIA, Collagen-induced arthritis; CON, Control; H&E, Hematoxylin and eosin; IHC, Immunohistochemistry *** p < 0.001.

COX7C, a subunit of cytochrome c oxidase (COX), plays a crucial role in mitochondrial energy production. Elucidating the function of COX7C may be associated with the metabolic and inflammatory pathways implicated in RA (35). As shown in **Supplementary Figures S9A, B**, COX7C demonstrated significant upregulation in most arthritis samples compared to HCs (p < 0.05). Subsequent ROC analyses revealed excellent diagnostic performance for distinguishing arthritis from HC (AUC = 0.913; **Supplementary Figure S9C**). Meanwhile, COX7C is expressed across various cell types (**Figures 6A, B**), including CD4+ T cells, CD8+ T cells, plasmablasts, and fibroblasts. Compared to normal PBMCs, COX7C expression was dysregulated in RA PBMCs, particularly in CD4+ T cell subsets such as central memory, effector memory, naïve thymus-derived CD4+ T cells, gamma-delta T cells, and memory B cells. Notably, elevated COX7C expression was observed in central memory CD4+ T cells, naïve thymus-derived CD4+ T cells, and gamma-delta T cells (**Figure 6C**, **Supplementary Table S17**). Furthermore, COX7C expression positively correlated with amino acid metabolism and TCA cycle metabolism in both the Binvignat et al. study (15) and the GSE109449 dataset (16) (**Figures 6D, E**). Through analyses of the GSE93776 dataset (14), the results revealed an upregulation of COX7C in central memory CD4+ T cells (p = 0.112) and effector memory CD4+ T cells (p = 0.111) in RA samples compared to HCs (**Figure 6F**). Additionally, eighteen drug compounds were identified as potential regulators of COX7C expression (adjusted p < 0.1; **Figure 6G**, **Supplementary Table S18**). Among these, 3′-Azido-3′-deoxythymidine, lobelin, clindamycin, ajmaline, azacyclonol, and dirithromycin exhibited high combined scores, as confirmed by comparison with drug sensitivity (**Supplementary Figures S9D, E**). Further investigation of the interactions between these compounds and COX7C were performed, the results demonstrated that dirithromycin exhibits potential as a therapeutic agent for RA using molecular docking analyses. Subsequently, CIA models were successfully established (**Figure 7A**). Histopathological examination of joint and synovial tissues was performed using H&E and Safranin O staining (**Figures 7B, C**). In the CON group, synovial tissues exhibited intact cartilage surfaces with no evidence of damage, and chondrocytes were arranged in a normal orientation. In contrast, the CIA group displayed pronounced synovial hyperplasia and extensive destruction of cartilage margins by proliferative synovial cells. IHC analyses revealed significantly elevated COX7C protein expression in the synovial tissues of CIA rats compared to the CON group (p < 0.05; **Figures 7D, E**). Collectively, these findings indicate that elevated COX7C expression is associated with metabolic dysregulation and may contribute to RA pathogenesis.

## 4 Discussion

Metabolic dysregulation has emerged as a critical pathogenic driver in RA (3, 6), with accumulating evidence revealing profound alterations in multiple metabolic pathways within synovial tissues and immune cells (4, 11). Although previous studies have reported changes in metabolite profiles throughout the course of RA (5, 36), several intermediate metabolites have been found to be either increased or decreased (5, 37). These metabolic shifts contribute to RA pathogenesis by directly or indirectly promoting inflammation and aberrant immune responses (6, 11). Multiple metabolic pathways, including glycolysis (38, 39), amino acid (2, 5), and TCA cycle (11), have been implicated in these perturbations, resulting in altered metabolic profiles in both immune and synovial cells during RA progression. Particularly, these metabolic changes described in the synovial microenvironment, where cells rely on glycolysis over oxidative phosphorylation despite its lower efficiency for ATP production, is known as the Warburg effect, also referred to as aerobic glycolysis (40, 41). Although glycolysis provides a more rapid production of ATP, which better supports the high energy demands of activated synovial immune cells (2, 10). In this study, the proportions of several immune cells were found to be significantly higher in RA samples using the CIBERSORT algorithm (21). Despite the inherent limitations of applying the LM22 signature to synovial tissue, the consistent identification of specific immune subsets and their validation through bulk RNA-seq of PBMCs and scRNA-seq confirm that these findings were reliable for our downstream analyses. Collectively, these results from complementary methodologies suggest that the upregulation of CD4+ memory T cells was associated with the pathogenesis of RA. As immune activity intensifies within the synovium and peripheral blood, the ensuing production of inflammatory mediators can spill into the circulation, contributing to the systemic manifestations of the disease (11, 41). Therefore, a comprehensive analyses of metabolic pathways is warranted to elucidate the relationship between cellular metabolism and immune infiltration in RA.

In this study, the impact of enrichment levels of the seven major metabolic pathways on the progression of RA was investigated. Notably, three dysregulated pathways, including TCA cycle, energy, and amino acid, were consistently associated with superior diagnostic potential in both PBMCs and synovial tissue from RA patients. Previous studies have reported increased lactic acid and decreased glucose levels in synovial fluid from RA patients (42), resulting from immune cell infiltration and metabolic dysregulation within the synovial microenvironment (11). Furthermore, altered expression of amino acid transporters, such as elevated glutamine transporters, has been observed in RA synovial cells, correlating with enhanced glutamine metabolism in activated immune cells (43). These changes are accompanied by broader metabolic alterations, including reduced mitochondrial respiration and impaired fatty acid oxidation, which notably affect the TCA cycle within both immune cells and their synovial microenvironment (11). In RA, CD4+ T cells become abnormally activated and infiltrate the synovium, where they interact with other immune cells such as macrophages and fibroblasts, leading to the production of pro-inflammatory cytokines that drive chronic inflammation and synovial tissue damage (44, 45). A positive correlation was observed between CD4+ T cells and dysregulation of the TCA cycle and amino acid metabolism in both PBMCs and synovial tissue, suggesting that CD4+ T cells undergo a metabolic shift and become increasingly dependent on these pathways to sustain their activation and function (11).

A set of eleven upregulated genes, including COX7C, COX7B, PDK1, PDK3, NDUFB8, COX11, NDUFAF4, SUCLG1, NDUFS1, PDHX, and SUCLA2, was identified in RA patients compared to HCs, and characterized as key hub genes with strong correlations to TCA cycle metabolism in RA. These genes are predominantly localized to the mitochondria and are involved in mitochondrial respiration. Notably, these identified hub genes encompass key players in the mitochondrial electron transport chain, such as COX7C, COX7B, and NDUFS1, indicating a broad dysregulation of mitochondrial oxidative phosphorylation in RA. Previous studies have demonstrated that immune cells such as macrophages and CD4+ T cells in RA patients exhibit impaired mitochondrial oxygen consumption and ATP production, along with increased generation of TCA cycle intermediates that contribute to various metabolic pathways (44, 46). Notably, these hub genes were positively correlated with CD4+ T cells, suggesting a role in supporting TCA cycle activity within these cells. While early RA often manifests as intermittent joint pain and stiffness with gradual onset, and established RA is marked by persistent and more severe symptoms, the expression patterns of these key metabolic genes remained similarly distinct across both stages, underscoring their potential diagnostic and mechanistic relevance throughout RA progression.

Subsequently, ten machine learning algorithms were applied to comprehensively evaluate the diagnostic potential of the key hub genes in RA. The LASSO model demonstrated the highest AUC value and showed robust performance across multiple GEO datasets, exhibiting strong diagnostic accuracy (AUC > 0.7) in both blood and synovial tissue samples, as well as in early and established stages of RA. The LASSO model values were positively correlated with the TCA cycle, CD4+ T cells, and the levels of COX7B or COX7C. A nomogram derived from the model translated multivariable gene expression data into actionable risk scores, with PDK1, COX7B, and COX7C contributing most significantly to the model. In RA patients, the LASSO model values showed significant positive correlations with CDAI, SJC28, D.VAS, and SDAI, which are widely used indicators of RA disease activity, reflecting chronic inflammation, joint pain, and functional impairment (1, 47). These correlations support the association between LASSO model values and disease severity, which is critical for guiding treatment strategies. RA patients with the absence of both RF and ACPA remain poorly understood and difficult to diagnose definitively (48). There were some emerging biomarkers offered some diagnostic value for this RA subtype (49), however, its distinguishing features are still largely unclear. Notably, our LASSO model demonstrated excellent diagnostic performance (AUC > 0.7) across multiple cohorts, indicating its potential superiority over traditional serological markers (RF or ACPA) in identifying RA. Elevated serum MMP3 levels are associated with increased disease activity and can serve as predictors of both disease progression and treatment response in RA (50). MMP3 activity can be induced by hypoxia-inducible factor (HIF)-1α, which upregulation in the synovial microenvironment is influenced by metabolic pathways (51). Moreover, metabolic alterations, particularly in glucose, lipid, and amino acid metabolism, are resulted in RA and are closely linked to MMP3 activity and the inflammatory cascade (9, 50).

This study challenges the prevailing notion that metabolic dysregulation across multiple cell types actively contributes to the progression of RA. Dysregulated metabolic pathways involving amino acids, lipids, carbohydrates, nucleotides, and TCA cycle were observed in the PBMCs of RA patients, with COX7B and COX7C showing positive correlations with these metabolic alterations. Notably, COX7C exhibited a strong correlation with fibroblasts in RA synovial tissue. Taken together, we proposed that COX7C might be associated with the regulation of TCA cycle in both blood and synovial tissue in RA. COX7C is a subunit of cytochrome c oxidase, which transfers electrons from cytochrome c to oxygen. Overexpression or deficiency of the COX7C subunit can affect the entire COX enzyme complex, leading to impaired metabolism and cellular dysfunction (52). Upregulation of COX7C has been observed in colon cancer and skin squamous cell carcinoma, where it is implicated in disease development and response to chemotherapy (52, 53). Additionally, in various metabolism-related diseases, elevated COX7C has been identified as a biomarker (54, 55), and single nucleotide variants in COX7C have been associated with an increased risk of Alzheimer’s disease (56). In this study, we observed COX7C enrichment in fibroblasts through analyses of GSE109449 dataset (16). Similarly, upregulation of COX7C was significantly increased in the synovial tissue of CIA rat models. Moreover, elevated COX7C expression was detected in CD4+ T cells, which were known to produce pro-inflammatory cytokines such as IL-6, IL-17, and TNF-α (57). Furthermore, CD4+ T cells in RA patients exhibit altered metabolic pathways, increased autophagy, and a tendency toward hyperproliferation and tissue invasion (58). Therefore, we believed that COX7C, like other COX subunits, might be linked to regulating metabolic pathways, such as TCA cycle and amino acid metabolism, in T cell activation, differentiation, and overall immune health (59).

Current treatments for RA primarily aim to alleviate symptoms, slow disease progression, and enhance quality of life (1, 60). While therapeutic advancements such as disease-modifying antirheumatic drugs (DMARDs), biologics, and JAK inhibitors have significantly improved RA management, however, these treatments showed limitations. A number of RA patients experience adverse effects, leading some to decline treatment due to safety concerns. Additionally, the high cost of biologics and JAK inhibitors often imposes substantial financial burdens. These challenges underscore the need for novel therapeutic strategies that are both effective and well-tolerated. In this study, we identified several drug compounds, such as 3′-Azido-3′-deoxythymidine, ajmaline, azacyclonol, and dirithromycin, that might target COX7C and modulate metabolic pathways in RA. While these compounds have not previously been reported as RA therapeutics, our findings suggest their potential role in disease treatment through metabolic regulation. Further investigation is warranted to validate their efficacy and safety in RA management.

This study has several limitations that should be acknowledged. First, while we found COX7C upregulation in the CIA model, the functional significance of this observation requires future investigation through genetic or pharmacological manipulation studies. Second, the proposed role of COX7C in RA requires functional validation through experiments involving COX7C perturbation in FLS or T cells, followed by an analyses of the resulting metabolic and cytokine profiles. Third, given the genetic heterogeneity of RA, the contributions of other differentially expressed genes to disease progression remain unexplored and should be addressed in future studies.

## 5 Conclusion

This study demonstrates that dysregulation of key metabolic pathways, particularly amino acid, energy, and the TCA cycle, play a crucial role in RA progression. Integrated bioinformatics and machine learning approaches identified eleven hub genes, including COX7C, COX7B, PDK1, PDK3, NDUFB8, COX11, NDUFAF4, SUCLG1, NDUFS1, PDHX, SUCLA2, that were implicated in metabolic reprogramming and immune infiltration. A LASSO logistic regression model incorporating these genes exhibited strong diagnostic performance in distinguishing RA patients from HCs across multiple datasets, with model scores correlating with clinical disease severity. Notably, COX7C emerged as a particularly important regulator, showing elevated expression in CD4+ T cell subsets and synovial fibroblasts, where it was strongly associated with TCA cycle, amino acid dysregulation, and immune infiltration patterns. Furthermore, several drug compounds with potential therapeutic value for RA by targeting COX7C were identified. Collectively, These findings highlight the key role of metabolic pathways in RA pathogenesis and provide new insights into potential diagnostic biomarkers and therapeutic strategies for RA management.

## Data Availability

The original contributions presented in the study are included in the article/**Supplementary Material**. Further inquiries can be directed to the corresponding authors.
