# Supplementary material for: Integrated analysis of the relationship between metabolic pathways and immune infiltration in rheumatoid arthritis
Source: Front Immunol. 2025 Dec 12;16:1679356. doi: 10.3389/fimmu.2025.1679356 (PMC12741155; doi:10.3389/fimmu.2025.1679356)
Supplement: Supplementary file 1 [file DataSheet1.pdf]

Supplementary Material

1 Supplementary Figures

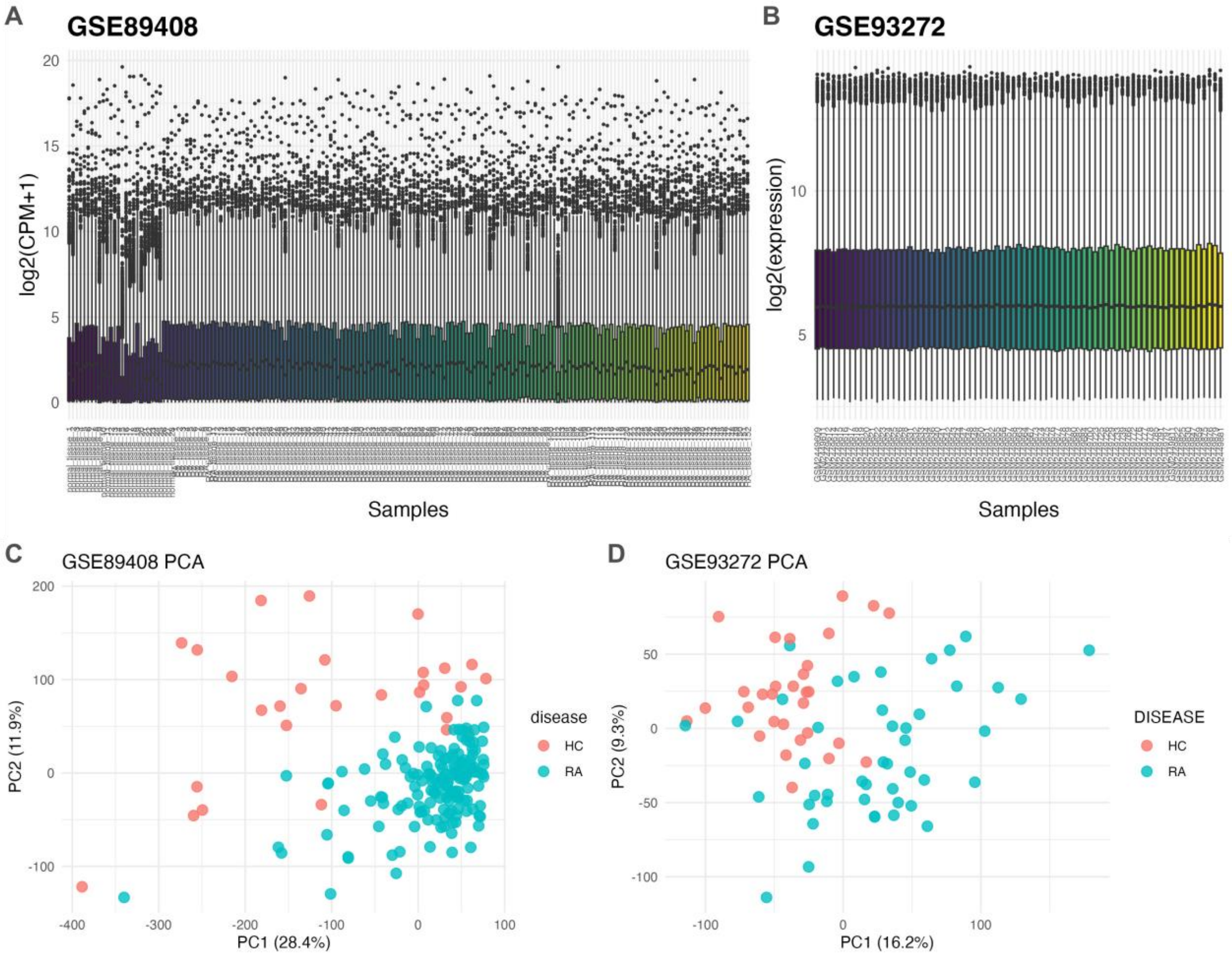

Supplementary Figure S1. The respective corrections for each dataset. Boxplot were validated in GSE89408 dataset **(A)** and GSE93272 datase **(B)**. PCA plots confirm the successful removal of batch effects both GSE89408 dataset **(C)** and GSE93272 datase **(D)**.

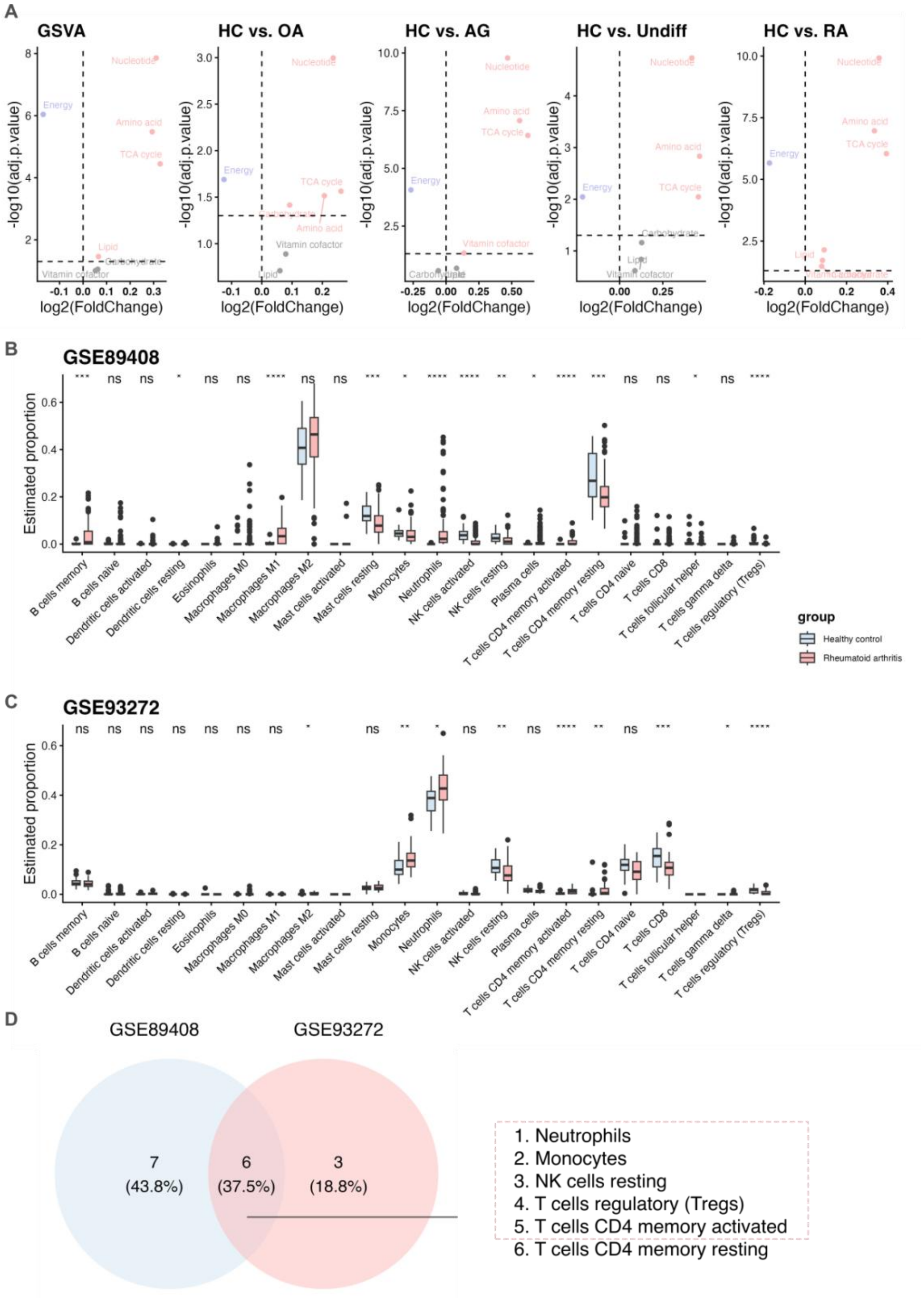

Supplementary Figure S2. The metabolic pathways, immune infiltration in RA. **(A)** Differential analysis of seven metabolic pathways across various arthritides compared to healthy controls was performed using the limma package on the GSE89408 dataset. CIBERSORT algorithm used to evaluate 22 types of immune cell infiltration, and comparison between the estimate proportion in 22 types of immune cell of RA samples and HCs in the GSE89408 dataset **(B)** and the GSE93272 dataset **(C)**. **(D)** Venn diagram showing the intersection of different immune cells between GSE89408 and GSE93272 datasets. CIBERSORT, cell type identification by estimating relative subsets of RNA transcripts; GSE, Gene Expression Omnibus Series; HC, healthy control; RA, rheumatoid arthritis. ****p* < 0.001; ***p* < 0.01; **p* < 0.05; ns, not significant.

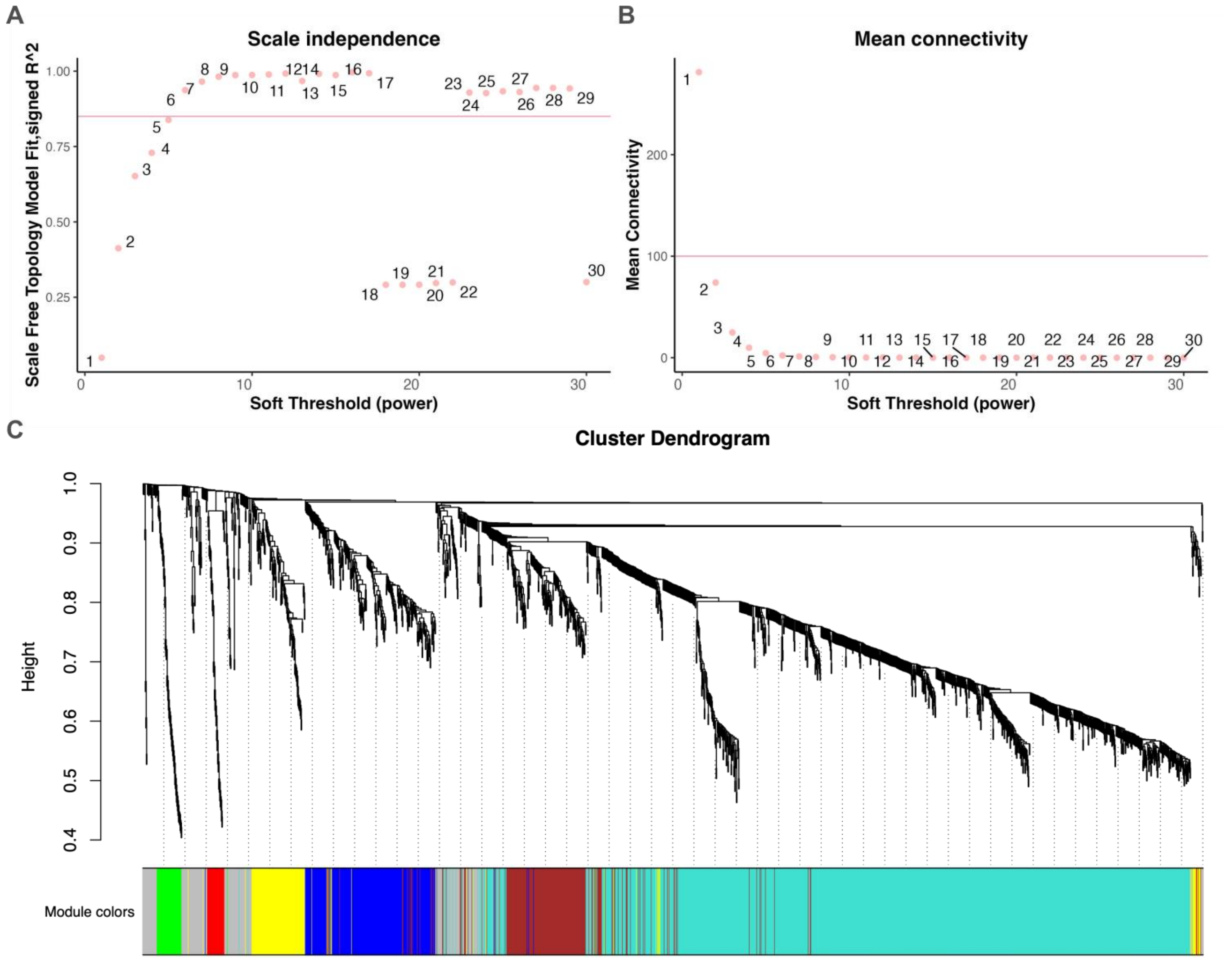

Supplementary Figure S3 WGCNA to mine differential gene modules. **A** Scale-independence of various soft-thresholding powers. **B** Mean connectivity analysis of various soft-thresholding powers. **C** Identification of co-expression modules.

A

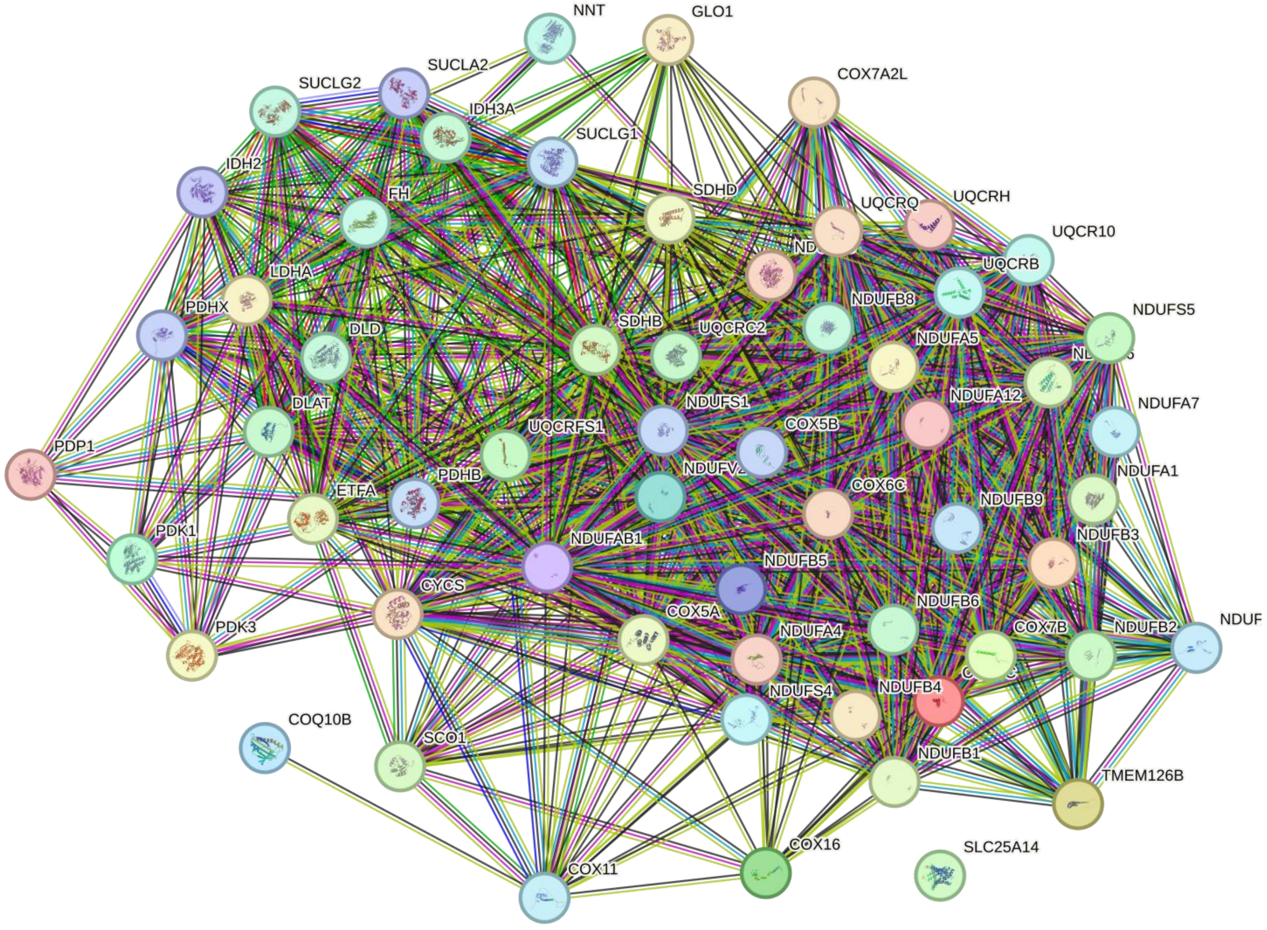

Supplementary Figure S4 PPI network showed based on STRING.org.

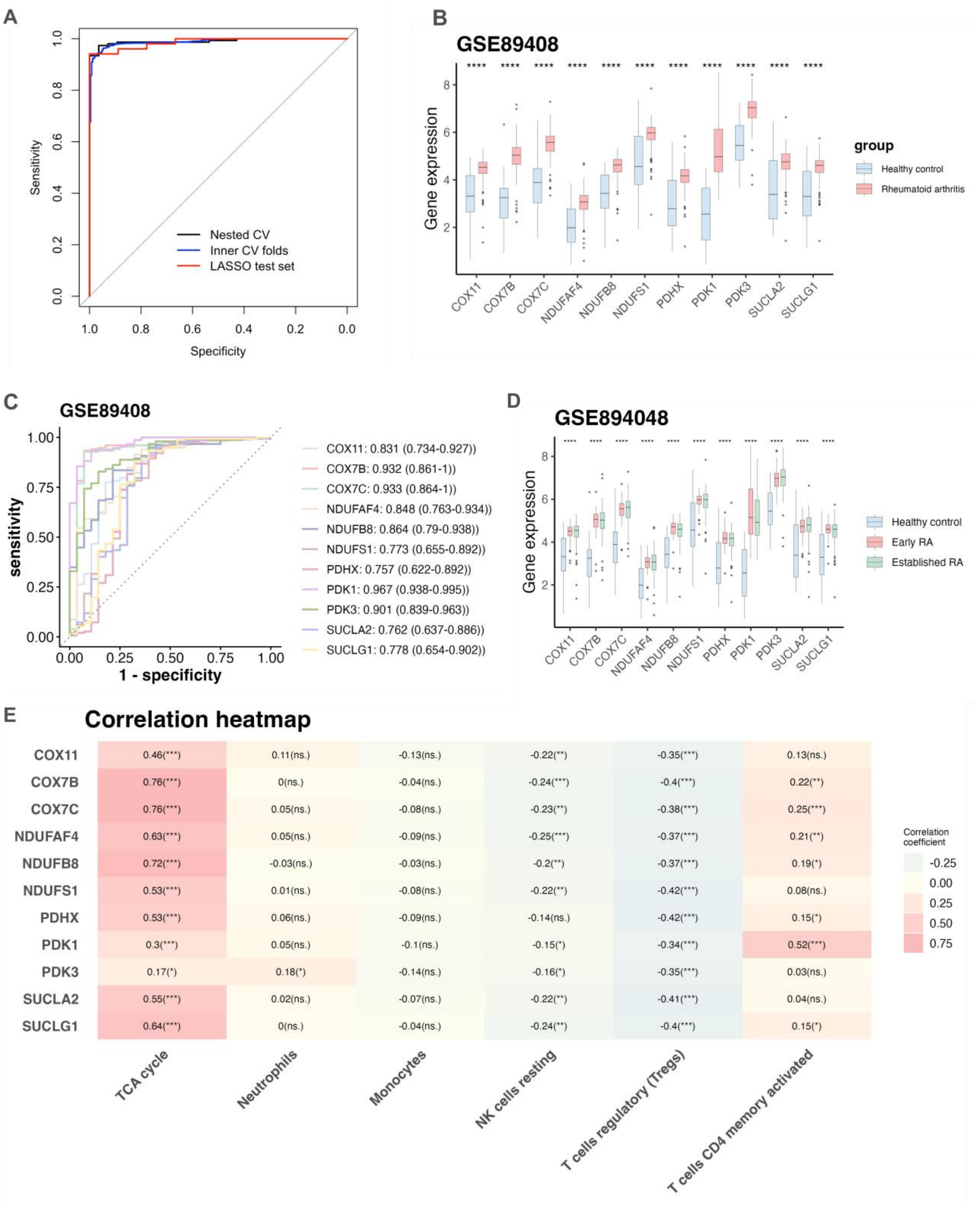

Supplementary Figure S5 Validation of Key Hub Genes Based on GSE89408 Dataset. **(A)** Comparison of Nested CV, inner CV folders, and LASSO model in ROC curve; **(B)** Expression differences of the eleven genes between RA and HCs; **(C)** ROC curves and corresponding AUC values were used to assess the predictive performance of these genes in distinguishing RA from HCs. **(D)** Expression differences of the eleven genes among early RA, established RA, and HCs; **(E)** Heatmaps showing the correlations between key hub genes and TCA cycle, immune cell infiltration. **Abbreviations:** AUC, area under the ROC curve; GSE, Gene Expression Omnibus Series; HC, healthy control; RA, rheumatoid arthritis; ROC, receiver operating characteristic; TCA, tricarboxylic acid. ***$p < 0.001$; **$p < 0.01$; *$p < 0.05$; ns, not significant.

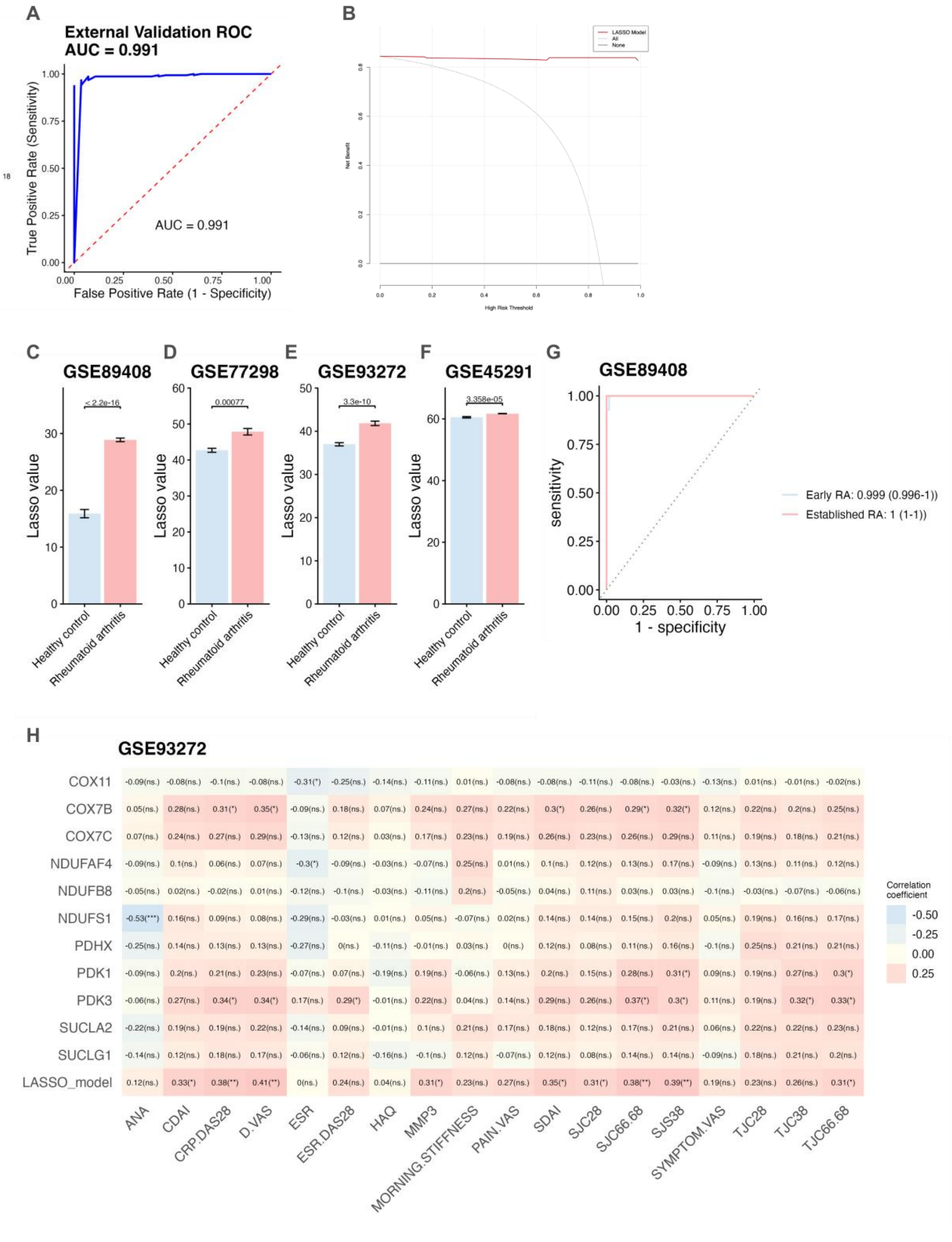

Supplementary Figure S6 Validation of LASSO model in multiple cohort. **(A)** External validation ROC curve; **(B)** Decision curve analysis.Expression differences of LASSO model values between RA and HCs, **(C)** GSE89408 dataset; (**D)** GSE77298 dataset; (**E)** GSE93272 dataset; **(F)** GSE45291 dataset. **(G)** ROC curves and corresponding AUC values were used to assess the predictive performance of LASSO model values in distinguishing RA from HCs. **(H)** Heatmaps showing the correlations between LASSO model values, key hub genes and clinical classification. **Abbreviations:** AUC, area under the ROC curve; GSE, Gene Expression Omnibus Series; HC, healthy control; LASSO, least absolute shrinkage and selection operator; RA, rheumatoid arthritis; ROC, receiver operating characteristic; TCA, tricarboxylic acid. ***p < 0.001; **p < 0.01; *p < 0.05; ns, not significant.

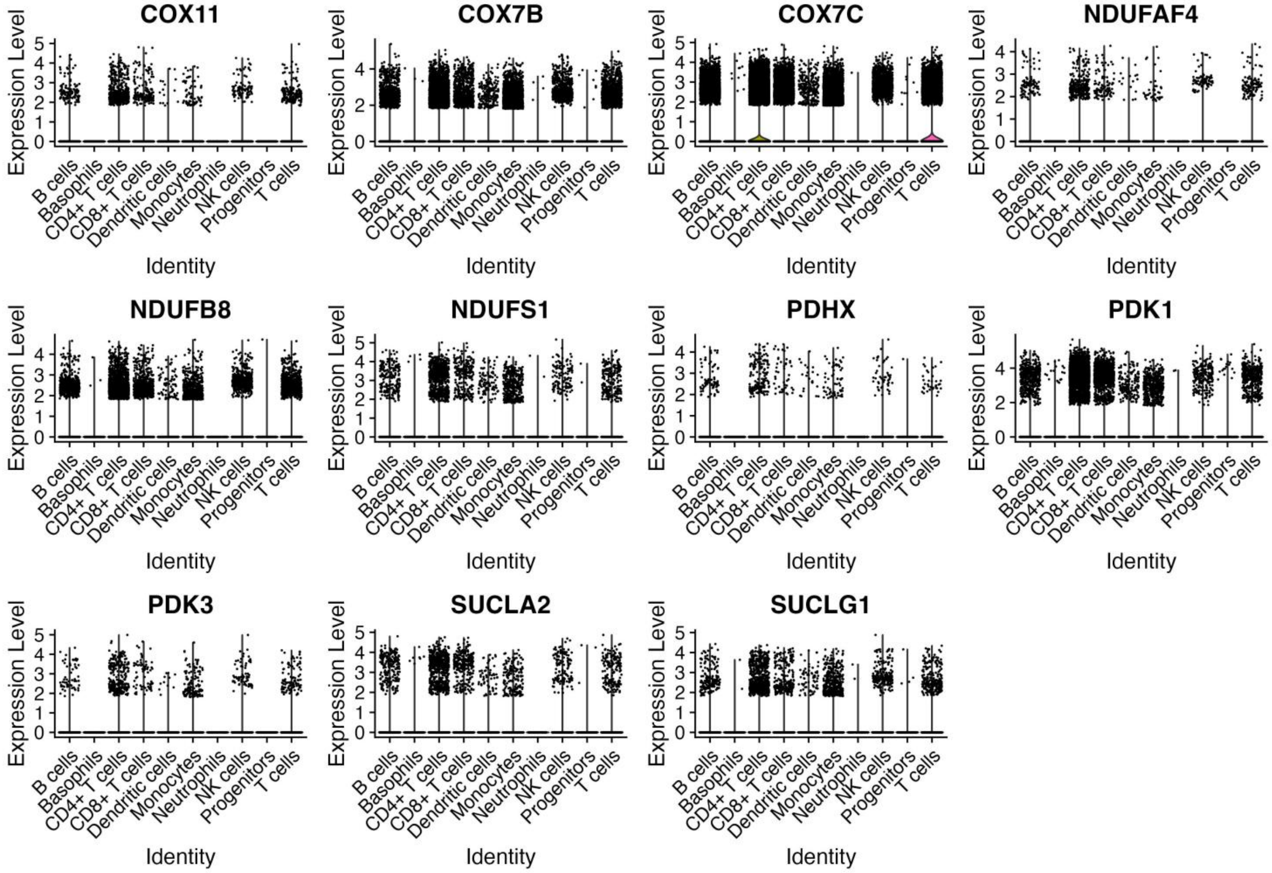

Supplementary Figure S7 Violin plot showing the distribution of key hub genes in different cell types.

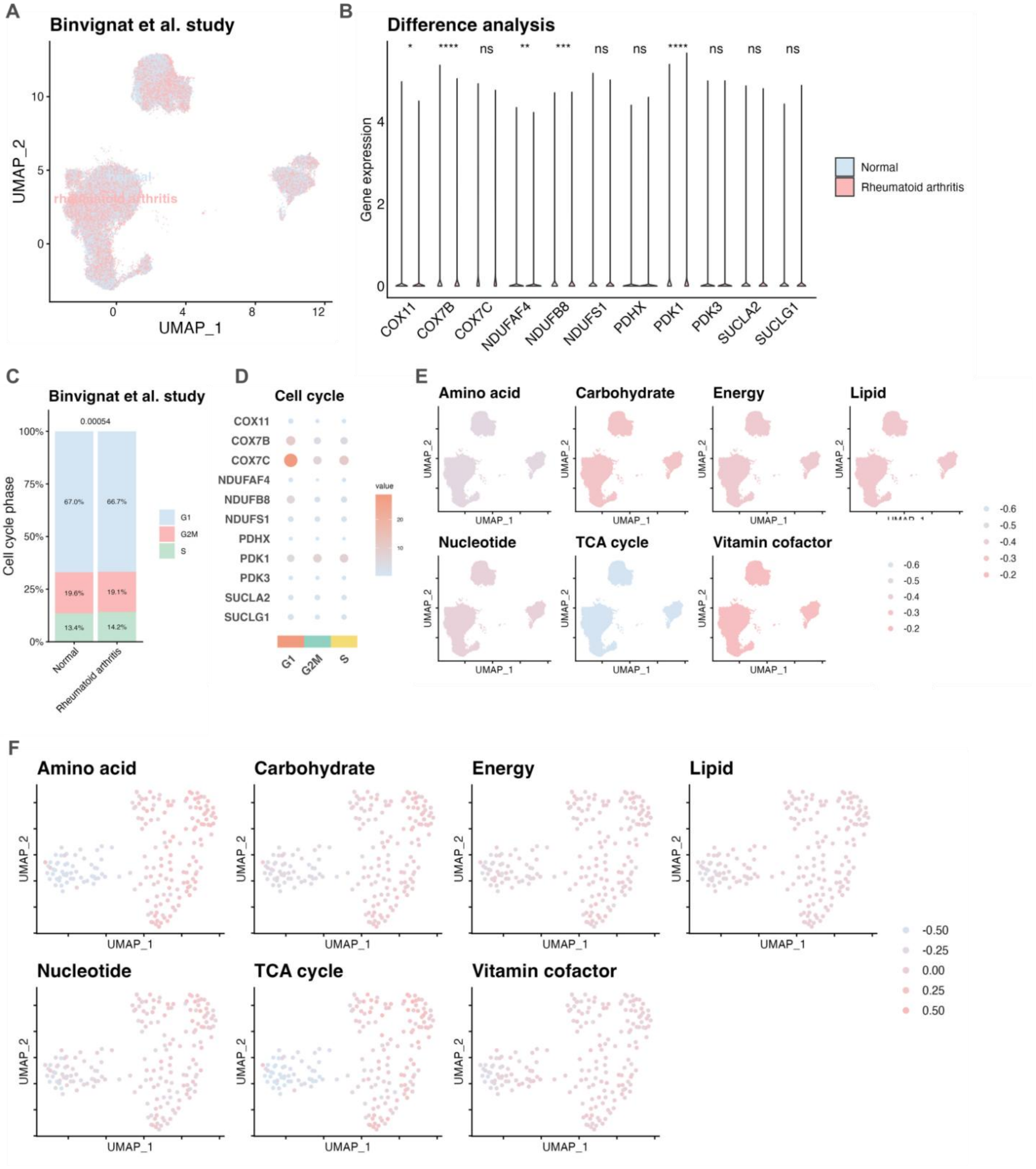

Supplementary Figure S8 Subcellular Localization Of Key Hub Genes. **(A)** UMAP plot showing the distribution of distinguishing RA from normals in PBMCs. **(B)** Expression differences of the eleven genes between RA and HCs. **(C)** Comparison of cell cycle proportions between RA and healthy samples in PBMCs. **(D)** Key hub genes in different stage of cell cycle.Key hub genes in different stage of cell cycle. **(E)** UMAP plot showing the distribution of different metabolic pathways in PBMCs. **(F)** UMAP plot showing the distribution of different metabolic pathways in fibroblast. **Abbreviations:** GSE, Gene Expression Omnibus Series; HC, healthy control; PBMC, Peripheral blood mononuclear cell; RA, rheumatoid arthritis. ***p < 0.001; **p < 0.01; *p < 0.05; ns, not significant.

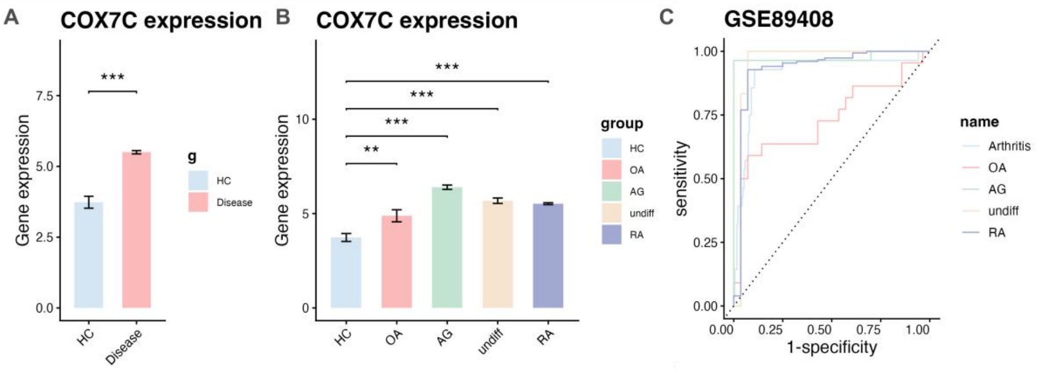

D

Ligand	Receptor	CDCKER_ENERGY（kcal/mol）	CDOCKER_INTERACTION_ENERGY（kcal/mol）
lobeline	5Z62	18.7272	-29.6342
clindamycin	5Z62	7.26964	-38.2275
dirithromycin	5Z62	6.8606	-70.334
ajmaline	5Z62	84.0826	-25.4677
azacyclonol	5Z62	-7.88582	-22.2577
3'-Azido-3'-deoxythymidine	5Z62	1.65882	-20.224

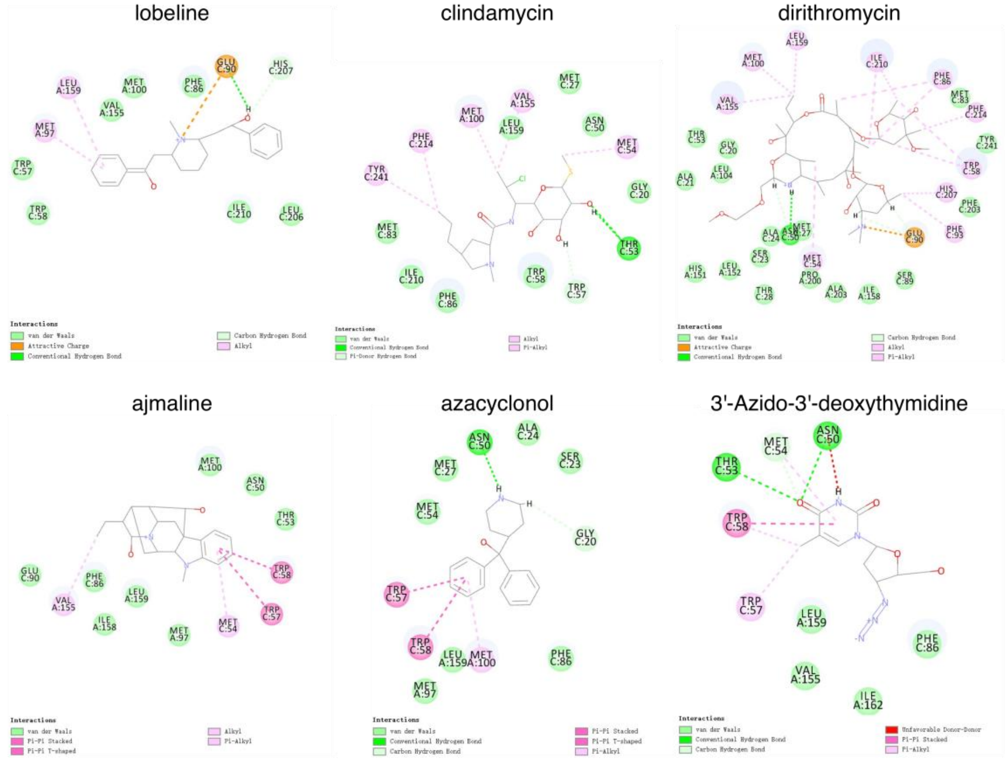

Supplementary Figure S9 The role of COX7C in RA and the prediction of potential compounds. **(A)** Comparative analysis between arthritis and healthy controls in GSE89408 dataset; **(B)** Comparative analysis across arthritis subtypes and healthy controls in GSE89408 dataset; **(C)** Predictive performance evaluation using ROC curves and AUC metrics; **(D)** Drug sensitivity analysis identified eighteen candidate compounds targeting COX7C; **(E)** Molecular docking analysis showing hydrogen bonding interactions between hub genes and the COX7C. **Abbreviations:** AG, arthralgia; GSE, Gene Expression Omnibus Series; HC, healthy control; OA, osteoarthritis; RA, rheumatoid arthritis; ROC, receiver operating characteristic; undiff, undifferentiated arthritis. ***$p < 0.001$; **$p < 0.01$.
